# Pyroptosis‐Inducing Biomaterials Pave the Way for Transformative Antitumor Immunotherapy

**DOI:** 10.1002/advs.202410336

**Published:** 2024-11-06

**Authors:** Hao Yin, Tanzhou Chen, Xiaoqu Hu, Wenting Zhu, Yida Li, Wenjie Sun, Lei Li, Hongmei Zhang, Qinyang Wang

**Affiliations:** ^1^ Institute for Advanced Research Wenzhou Medical University Wenzhou Zhejiang 325027 P. R. China; ^2^ Department of Oncology Xijing Hospital of Air Force Military Medical University Xi'an Shaanxi 710032 P. R. China; ^3^ The First Affiliated Hospital of Wenzhou Medical University Wenzhou Medical University Wenzhou Zhejiang 325027 P. R. China; ^4^ Department of Radiation and Medical Oncology Wenzhou Key Laboratory of Basic Science and Translational Research of Radiation Oncology Zhejiang Engineering Research Center for Innovation and Application of Intelligent Radiotherapy Technology The Second Affiliated Hospital and Yuying Children's Hospital of Wenzhou Medical University Wenzhou Zhejiang 325027 P. R. China; ^5^ The First Hospital of Xi'an Jiaotong University Xi'an Shanxi 710061 P. R. China

**Keywords:** antitumor immunotherapy, biomaterials, gasdermin, pyroptosis

## Abstract

Pyroptosis can effectively overcome immunosuppression and reactivate antitumor immunity. However, pyroptosis initiation is challenging. First, the underlying biological mechanisms of pyroptosis are complex, and a variety of gasdermin family proteins can be targeted to induce pyroptosis. Second, other intracellular death pathways may also interfere with pyroptosis. The rationally designed gasdermin protein‐targeting biomaterials are capable of inducing pyroptosis and have the capacity to stimulate antitumor immune function in a safe and effective manner. This review provides a comprehensive overview of the design, function, and antitumor efficacy of pyroptosis‐inducing materials and the associated challenges, with a particular focus on the design options for pyroptosis‐inducing biomaterials based on the activation of different gasdermin proteins. This review offers a valuable foundation for the further development of pyroptosis‐inducing biomaterials for clinical applications.

## Graphical abstract

Pyroptosis plays a role in activating antitumour immunity; however, tumours have evolved mechanisms to evade pyroptosis. The development of pyroptosis‐inducing biomaterials is a significant factor in the advancement of pyroptosis‐driven transformative antitumour therapeutics. In this review, Qinyang Wang, Hongmei Zhang, and Lei Li, et al. provide a comprehensive summary of the latest research advances in pyroptosis‐inducing biomaterials, offering valuable perspectives.

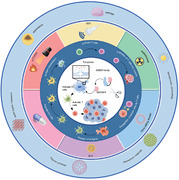

## 1 Introduction

The development of antitumor immunotherapy is a revolutionary advance in modern medicine and has become an important component of cancer treatment.^[^ ^1^ ^]^ The fundamental premise of antitumor immunotherapy is to enhance the ability of the immune system to effectively recognize and attack malignant cells, thereby eradicating them.^[^ ^2^ ^]^ Immune checkpoint inhibitors, such as monoclonal antibodies programmed death 1 antibody (PD‐1), programmed cell death‐Ligand 1 (PD‐L1), and cytotoxic T lymphocyte‐associated antigen‐4 (CTLA‐4) antibodies are considered representative Immunotherapeutic agents.^[^ ^3^ ^]^ These inhibitors have demonstrated considerable efficacy in the treatment of a range of solid tumors and haematological malignancies.^[^ ^4^ ^]^ Moreover, they have demonstrated remarkable efficacy in refractory or advanced cases, resulting in prolonged survival and, in some instances, a complete cure. Nevertheless, the efficacy of immune checkpoint inhibitors remains limited, as ≈70% of patients do not benefit from these agents.^[^ ^5^ ^]^ In recent years, as our understanding of the mechanisms of tumor immune evasion due to patient heterogeneity has increased, researchers have shown that during tumorigenesis and progression, tumor cells often evade the surveillance of the host immune system through various mechanisms, thereby creating an immunosuppressive microenvironment conducive to their growth, which leads to the failure of antitumor immunotherapy.

Pyroptosis is a form of programmed cell death that has recently been a popular research topic because of its antitumor immune function. In contrast to conventional apoptosis, pyroptosis is characterized by pronounced cell swelling, plasma membrane rupture, and the massive release of cellular contents. These cellular contents include including various inflammatory cytokines that activate the immune system.^[^ ^6^ ^]^ This distinctive process is of pivotal importance in the regulation of the tumor microenvironment. Studies have revealed that signaling molecules and damage‐associated molecular patterns (DAMPs) released during pyroptosis, including high mobility group protein B1 (HMGB1), adenosine triphosphate (ATP), and IL‐1 family cytokines, can recruit immune cells to infiltrate tumors and activate them.^[^ ^7^ ^]^ In 2020, three pioneering studies collectively established that pyroptosis can enhance the antitumor immune response.^[^ ^8^ ^]^ These findings indicate that tumor cell‐specific pyroptosis can eliminate tumors and prevent tumor recurrence in preclinical models. However, gasdermin, the protein that executes pyroptosis, is commonly downregulated in tumor cells, thus enabling their evasion of pyroptosis. Additionally, excessive and nontumor cell‐specific pyroptosis leads to cytokine release syndrome, which can be life‐threatening. These issues have impeded the further development of pyroptosis‐mediated antitumor immunotherapy.

There are numerous potential applications of biomaterials whose physical and chemical properties can be precisely controlled in the fields of tissue engineering, drug delivery and disease treatment.^[^ ^9^ ^]^ Consequently, investigating pyroptosis induction in tumor cells via tumor‐specific biomaterials may provide a novel approach to antitumor immunotherapy. For example, the delivery of pyroptosis inducers via biomaterials with tumor‐targeting capabilities can result in tumor cell‐specific pyroptosis.^[^ ^10^ ^]^ Furthermore, some biomaterials themselves can trigger pyroptosis.^[^ ^11^ ^]^ Consequently, the development of pyroptosis‐inducing biomaterials that can induce an antitumor immune response is of paramount importance for enhancing the efficacy of tumor therapy. This is particularly the case with malignant tumors that have demonstrated resistance or ineffectiveness to conventional therapies. However, biomaterial‐mediated activation of pyroptosis must adhere to basic biological principles; otherwise, there is a high likelihood that apoptosis will be activated or that other types of cell death with similar morphological features will be observed (e.g., ferroptosis).^[^ ^11^ ^]^ Therefore, elucidating the mechanisms of pyroptosis for the development of biomaterials and enhancement of immunotherapy is important.

**Figure 1:**
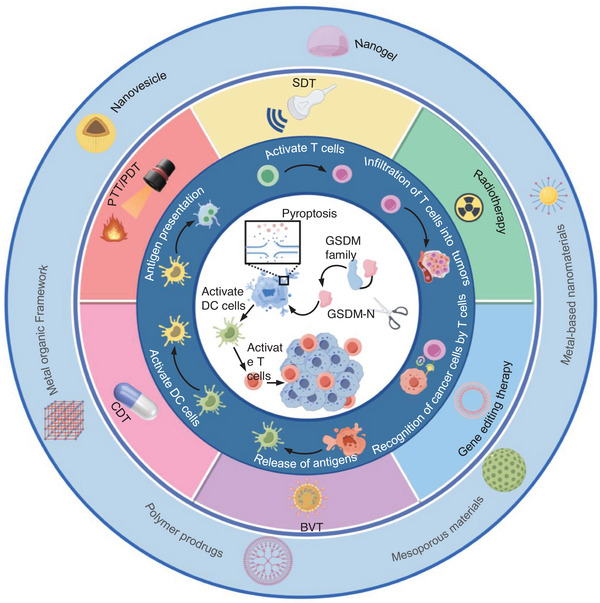
The schematic diagram of activating GSDM family proteins to mediate cancer cell pyroptosis and enhance antitumor immunity under different induction strategies of different biomaterials. The activation of GSDM family protein‐mediated pyroptosis can be achieved through the combination of various biomaterials with different types of therapeutic modalities, including photothermal therapy (PTT), photodynamic therapy (PDT), chemodynamic therapy (CDT), sonodynamic therapy (SDT), engineered bacterial or viral therapy (BVT), gene editing therapy, and radiotherapy. In general, pyroptosis is induced to release a large number of inflammatory factors and tumor‐associated antigens, which activate dendritic cells (DCs). The antigen‐presenting function of DCs is then used to activate T cells, enhance the infiltration of T cells in the tumor site, and specifically kill tumor cells. It is possible to initiate a robust antitumor immune response that will result in the complete eradication of tumors. The picture was drawn by Figdraw.

This review provides a comprehensive summary of biomaterials developed for pyroptosis‐mediated antitumor immunomodulation. Given that gasdermin proteins are the executors of pyroptosis and that the upstream activation signals of this family of proteins vary, this review focuses on the ability of biomaterials to activate different gasdermin (GSDM) proteins, as well as the underlying mechanisms by which these biomaterials initiate antitumor immunity through the targeting of gasdermin proteins (**Figure** **1**). In particular, we begin by reviewing the process of pyroptosis, emphasizing that the release of diverse inflammatory mediators during pyroptosis facilitates immune cell infiltration within the tumor and elicits a robust antitumor immune response. Second, an examination of the current state of research on the GSDM protein will be conducted, in addition to an analysis of the molecular mechanisms underlying the activation of the GSDM protein. Furthermore, we highlight recent advances in tumor immunotherapy that involves the activation of cancer cell pyroptosis using various bioresponsive materials.

## 2 Gasdermin Proteins Serve as the Final Executors of Pyroptosis

**Figure 2:**
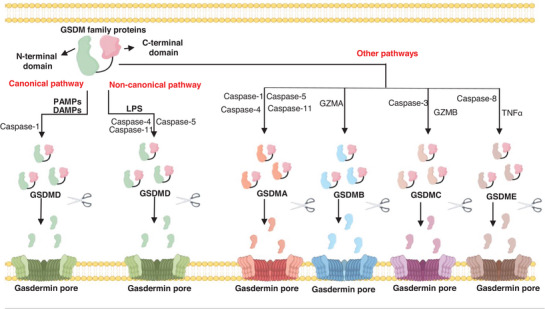
The mechanism of gasdermin protein activating N‐terminal membrane insertion and pore formation. Pyroptosis is a programmed cell death mediated by GSDM protein. The N‐terminal of GSDM protein is inhibited under normal physiological conditions. When this inhibition is destroyed, such as caspase‐1/3/4/5/8/11 are activated, GSDM protein will be cleaved to release the N‐terminal of GSDM protein. The accumulation of the N‐terminus of GSDM protein on the cell membrane will form membrane pores and lead to pyroptosis. The picture was drawn by Figdraw.

The gasdermin protein family was identified in the early 21st century as potential candidates responsible for various alopecia‐like skin mutations in mice.^[^ ^12^ ^]^ Initial studies highlighted the strong sequence similarity between the N‐terminal region of gasdermins and the deafness autosomal dominant non‐syndromic sensorineural protein 5 (DFNA5).^[^ ^13^ ^]^ This homology led to the identification of several additional gasdermin family members and gasdermin‐like proteins, which currently include gasdermin A (GSDMA), gasdermin B (GSDMB), gasdermin C (GSDMC), gasdermin D (GSDMD), and gasdermin E (GSDME).^[^ ^6^ ^]^ Following the discovery of gasdermin‐like proteins, subsequent studies established a connection between these proteins and cell viability as well as inflammation. However, the role of gasdermins in the cell death program and the specific types of cell death regulated by these proteins remain uncertain. At the time of the discovery of the gasdermin protein, researchers also observed pyroptosis occurring in macrophages following bacterial infection or exposure to bacterial toxins. Initially, it was believed that this form of cell death was specific to macrophages and dependent on caspase‐1, as caspase‐1 is responsible for cleaving the pro‐inflammatory cytokine interleukin‐1β (IL‐1β).^[^ ^14^ ^]^ However, subsequent studies demonstrated that cytoplasmic pattern recognition receptors (PRRs) can recognize pathogen‐associated molecular patterns (PAMPs) or DAMPs, leading to the formation of inflammasomes.^[^ ^14^ ^d,^ ^15^ ^]^ These inflammasomes recruit and activate caspase‐1, triggering pyroptosis.^[^ ^16^ ^]^ Additionally, murine caspase‐11 and human caspase‐4/5 act directly as PRRs, recognizing bacterial lipopolysaccharide lipid A, triggering the assembly of inflammasomes, and ultimately inducing pyroptosis.^[^ ^17^ ^]^ This discovery challenged the conventional understanding of inflammasomes. Moreover, the discovery of the noncanonical pathway of inflammasome activation revealed that not only caspase‐1 activation but also inflammatory caspase activation can induce pyroptosis.^[^ ^18^ ^]^ Nonetheless, the specific mechanism underlying pyroptosis induced by inflammatory caspase activation remains unclear. In 2015, two independent studies revealed the same protein known as GSDMD. Dixit et al. screened chemically mutagenized mouse mutants and demonstrated that GSDMD is involved in the lipopolysaccharide (LPS)‐induced activation of inflammasomes via the noncanonical pathway.^[^ ^17a^ ^]^ Shao et al. utilized newly established CRISPR‐Cas9 technology to analyze caspase‐11‐ and caspase‐1‐induced pyroptosis in cell lines and identified GSDMD as the substrate for all inflammatory caspases, serving as the primary executor of pyroptosis.^[^ ^17d^ ^]^ Consequently, these two studies revealed the molecular mechanisms underlying pyroptosis, defining it as programmed cell death mediated by gasdermin proteins (**Figure** **2**)

GSDMD is a 480‐amino acid protein consisting of N‐terminal and C‐terminal domains. In vitro studies have demonstrated that GSDMD is the sole mediator of cell death via the noncanonical inflammasome pathway. The induction of pyroptosis mediated by GSDMD has been well studied and can occur through the classical pathway and nonclassical pathway. The classical pathway relies mainly on the activation of the NOD‐like receptor (NLR) family (NLRP1, NLRP3, NLRC4, and AIM2). This process typically occurs when PAMPs or DAMPs, such as bacterial LPS, nucleic acids, and ATP, are detected within cells. Upon recognition of these signals by intracellular PRRs, the assembly of the NLRP3 inflammasome is triggered. Once assembled, the NLRP3 inflammasome activates the effector enzyme ASC, which, in turn, activates and cleaves Pro‐caspase‐1 to generate active caspase‐1. Activated caspase‐1 serves two main functions. It cleaves GSDMD to produce a peptide containing the GSDM‐NT active domain, leading to cell membrane perforation, cell rupture, the release of cellular contents, and the initiation of an inflammatory response. Concurrently, it cleaves the precursors of IL‐1β and interleukin‐18 (IL‐18), resulting in the formation of active IL‐1β and IL‐18, which are subsequently released extracellularly to recruit inflammatory cells, thereby amplifying the inflammatory response. In the nonclassical pathway, when cells are stimulated with LPS, caspase‐4, caspase‐5, and caspase‐11 can directly bind to LPS and initiate pyroptosis. Activated caspase‐4/5/11 cleaves the GSDMD protein, with the N‐terminus of GSDMD mediating cell membrane dissolution and pyroptosis. Additionally, activated caspase‐4/5/11 also induces NLRP3 inflammasome activation, which in turn activates caspase‐1, ultimately leading to the production and release of IL‐1β and IL‐18.^[^ ^17^, ^19^ ^]^ The N‐terminus of GSDMD is crucial for inducing pyroptosis, with the oligomerized GSDMD‐N‐terminal protein forming molecular pores in the membrane. These pores directly disrupt the cell membrane, leading to severe leakage and the lysis of biofilms, which is similar to bacterial toxins that punch holes in the cell membrane. High‐resolution atomic force microscopy (AFM) analysis has further elucidated the dynamic process of GSDMD‐N‐terminal protein intercalation, polymerization, and pore assembly. These pores compromise the cellular osmotic pressure, causing cell swelling and lysis, which are characteristic biochemical manifestations of pyroptosis.^[^ ^17^, ^20^ ^]^

GSDME was initially identified as a gene linked to nonsyndromic hearing loss in humans. It belongs to the gasdermin family of proteins and contains a characteristic caspase‐3 cleavage site between its N‐terminal and C‐terminal domains.^[^ ^18^, ^21^ ^]^ This caspase‐3 cleavage site enables activation of GSDME through caspase‐3 cleavage, leading to the induction of pyroptosis. Certain chemotherapeutic agents, such as doxorubicin, cisplatin, and etoposide, have been demonstrated to induce pyroptosis in cell lines that express high levels of GSDME but induce apoptosis in GSDME‐negative cells. In light of the role of gasdermin family proteins in facilitating pyroptosis and the observation that pyroptosis does not rely solely on the activity of inflammatory caspases, Shao et al. proposed to redefine pyroptosis as a form of programmed cell death mediated by gasdermin.^[^ ^21a^ ^]^ This reinterpretation has opened novel avenues for investigating cell death and inflammatory immune responses.

GSDMA is expressed primarily in epithelial cells, particularly in skin and digestive tract tissues. However, its expression is suppressed in related tumor cells.^[^ ^12b^ ^]^ Normally, GSDMA exists as an inactive precursor.^[^ ^22^ ^]^ When exposed to specific stimuli, such as infection or inflammatory signals, PRRs are activated through a sequence of signal transduction events. These events ultimately lead to the activation of inflammatory caspases from the caspase family, including caspase‐1, ‐4, ‐5, or ‐11. The activated caspases selectively cleave the N‐terminal domain of GSDMA, liberating a fragment with transmembrane activity. This released N‐terminal fragment is then integrated into the cell membrane, forming a pore with a diameter measuring ≈10–30 nm. Consequently, intracellular substances leak out, causing cell swelling and eventual rupture, which ultimately triggers pyroptosis.^[^ ^23^ ^]^

The GSDMB protein is a newly discovered member of the GSDM protein family. Its involvement in the progression of various types of cancer has been revealed, and its expression is increased in liver cancer, cervical cancer, breast cancer, and gastric cancer.^[^ ^24^ ^]^ Nonetheless, the exact role of GSDMB in the development of tumors in vivo remains inconclusive, despite in vitro experiments demonstrating that its elevated expression leads to pyroptosis. Recent studies have revealed that lymphocyte‐derived granzyme A (GZMA) is capable of cleaving GSDMB and inducing pyroptosis.^[^ ^25^ ^]^

GSDMC is a relatively poorly understood GSDM protein. Initially, identified as melanoma‐derived leucine zipper‐containing extranuclear factor (MLZE), it was found to be highly expressed in metastatic melanoma cells.^[^ ^26^ ^]^ Further investigations revealed that GSDMC undergoes specific cleavage by caspase‐8 upon treatment with tumor necrosis factor‐α (TNFα), leading to the cleavage of the N‐terminal domain of GSDMC. This cleavage event results in the formation of pores on the cell membrane and the induction of pyroptosis. Recent reports have linked high expression levels of GSDMC to poor prognosis and impaired immune cell infiltration in pancreatic ductal adenocarcinoma, supporting its role in promoting cancer.^[^ ^27^ ^]^ However, contrasting findings have shown inhibited GSDMC expression in oesophageal squamous cell carcinoma, suggesting its potential as a tumor suppressor gene.^[^ ^28^ ^]^ The true role of GSDMC in cancer development remains unclear and requires further investigation.

## 3 Gasdermin‐Targeting Biomaterials Induce Pyroptosis

As nanotechnology and immunotherapy have advanced, interest among researchers in increasing the efficacy of tumor immunotherapy through the induction of pyroptosis has increased.^[^ ^6^, ^29^ ^]^ This chapter delves into the intricate mechanisms by which the cleavage of distinct GSDM family proteins is triggered to enhance the efficacy of antitumor immunity (**Table** **1**). We provide an in‐depth review of various biological platforms, including photothermal therapy (PTT), photodynamic therapy (PDT), dynamic therapy (CDT), sonodynamic therapy (SDT), and engineered bacterial or viral therapy (BVT), that activate these GSDM proteins to initiate pyroptosis, thereby contributing to innovative strategies for combating malignancies.

**Table 1:** Review of gasdermin‐targeting pyroptosis‐inducing biomaterials.

Target	Biomaterials	Therapy	Molecular mechanism	Cancer type	References
GSDMD	VB12‐sericin‐PBLG‐IR780	PTT/PDT	caspase‐1	Gastric cancer	[30]
GSDMD	DPITQ	PTT/PDT	caspase‐1	Breast cancer	[31]
GSDMD	TBD‐R PSs	PDT	caspase‐1	Breast, Cervical cancer and Glioblastoma	[32]
GSDMD	OA@IR820	PDT	caspase‐1	Malignant melanoma	[33]
GSDMD	Pt1 and Pt2	PDT	caspase‐1	Cervical cancer	[34]
GSDMD	CA‐Re	PDT	caspase‐1	Breast cancer	[35]
GSDMD	AIE	PDT	caspase‐1	pancreatic cancer	[36]
GSDMD	TFL	PDT	caspase‐1	Breast cancer	[37]
GSDMD	ChS‐Ce6 nanovesicles	PDT	caspase‐1	Lung cancer	[38]
GSDMD	DOX/Ce6‐OMVs@M	PDT	caspase‐1	Breast cancer	[39]
GSDMD	Ir‐HEcN	PDT	caspase‐1	Breast cancer	[40]
GSDMD	Ac‐DEVDD‐TPP	PDT	caspase‐1	Squamous cell carcinoma	[41]
GSDMD	D1	PDT	caspase‐1	Breast cancer	[42]
GSDMD	YBS‐BMS NPs‐RKC	PDT	caspase‐1	Prostate cancer	[43]
GSDMD	NiS2/FeS2	PDT	caspase‐1	Breast cancer	[44]
GSDMD	Mito‐ZS, Lyso‐ZS and ER‐ZS	PDT	caspase‐1	Breast cancer	[45]
GSDMD	ZPHM	PDT	caspase‐1	Breast cancer	[46]
GSDMD	Cu‐TBB	PDT	caspase‐1	Breast cancer	[47]
GSDMD	CTEP	PDT	caspase‐1	Liver cancer	[48]
GSDMD	PNSO NPs	CDT	caspase‐1	Breast cancer	[49]
GSDMD	Lip‐MOF	CDT	caspase‐1	Cervical cancer	[50]
GSDMD	Tf‐LipoMof@PL	CDT	caspase‐1	Breast cancer	[51]
GSDMD	PTAVs	CDT	caspase‐1	Breast cancer	[52]
GSDMD	NaCl@ssss‐VHMS	CDT	caspase‐1	Liver cancer	[53]
GSDMD	CRS	CDT	caspase‐1	Glioblastoma	[54]
GSDMD	LZnO_2_	CDT	caspase‐1	Breast cancer	[55]
GSDMD	ZIF‐8	CDT	caspase‐1	Breast cancer	[56]
GSDMD	NaHCO_3_ NPs	CDT	caspase‐1	Breast cancer	[57]
GSDMD	Na_2_S_2_O_8_	CDT	caspase‐1	Breast cancer	[58]
GSDMD	CQG NPs	CDT	caspase‐1	Breast cancer	[59]
GSDMD	CSSG	CDT	caspase‐1	Breast cancer	[60]
GSDMD	TSC	CDT	caspase‐1	Liver cancer	[61]
GSDMD	Cu_2_(PO_4_)(OH) NPs	CDT	caspase‐1	Colon cancer	[62]
GSDMD	BEM NPs	CDT	caspase‐1	Breast cancer	[63]
GSDMD	Al@P‐P	CDT	caspase‐1	Breast cancer	[64]
GSDMD	Cu‐N4 SA	CDT	caspase‐1	Breast cancer	[65]
GSDMD	LaCoO_3_	SDT	caspase‐1	Breast cancer	[66]
GSDMD	LFO@Gox	SDT	caspase‐1	Breast cancer	[67]
GSDMD	hMnO_2_ NPs	SDT	caspase‐1	Malignant melanoma	[68]
GSDMD	VNP‐GD	BVT	caspase‐1	Ovarian cancer	[69]
GSDMD	LPZ	BVT	caspase‐1	Breast cancer	[70]
GSDMD	RCA	BVT	caspase‐1	Breast cancer	[71]
GSDMD	Fe(II)‐H_2_O_2_‐Fe(III)	BVT	caspase‐1	Colon cancer	[72]
GSDME	As_2_O_3_‐NPs	Chemotherapy	caspase‐3	Liver cancer	[73]
GSDME	HP‐DOX/JQ1	Chemotherapy	caspase‐3	Breast cancer	[74]
GSDME	MCPP	Chemotherapy	caspase‐3	Colon cancer	[75]
GSDME	PDNP	Chemotherapy	caspase‐3	Colon cancer	[76]
GSDME	(SAS/DOX@OHS‐PEG)	Chemotherapy	caspase‐3	Breast cancer	[77]
GSDME	BIK system	Chemotherapy	caspase‐3	Breast, Colon cancer	[78]
GSDME	Pt‐In NP	Chemotherapy	caspase‐3	Pancreatic cancer	[79]
GSDME	COF‐909‐Cu	PTT/CDT	caspase‐3	Breast cancer	[80]
GSDME	COF919	PTT/PDT	caspase‐3	Breast cancer	[81]
GSDME	TPy‐imine COF and TPy‐vinyl COF	PTT/PDT	caspase‐3	Breast cancer	[82]
GSDME	I‐L@NM	PTT	caspase‐3	Colon cancer	[83]
GSDME	ANPS	PDT	caspase‐3	Breast, Lung cancer	[10]
GSDME	NI‐TA	PDT	caspase‐3	Breast cancer	[84]
GSDME	R@IrP	PDT	caspase‐3	Malignant melanoma	[85]
GSDME	HCS‐FeCu	PDT	caspase‐3	Breast cancer	[86]
GSDME	NP2	PDT	caspase‐3	Breast cancer	[87]
GSDME	L@NBMZ	PDT	caspase‐3	Breast cancer	[88]
GSDME	CaNMs	CDT	caspase‐1	Breast cancer	[89]
GSDME	aDeFer‐2	CDT	caspase‐1	Malignant melanoma	[90]
GSDME	CLNDN	CDT	caspase‐1	Colon cancer	[91]
GSDME	ORFV	BVT	caspase‐1	Breast, Colon cancer	[92]
GSDME	MPNPs	BVT	caspase‐1	Breast cancer	[93]
GSDME	HVMNVs@Fe‐C	BVT	caspase‐1	Malignant melanoma	[94]
GSDMA	Phe‐BF_3_	Bioorthogonal chemical	GSDMA^NT^	Breast cancer	[8a]
GSDMB	GSDMB^NT^ mRNA@LNPs	mRNA	GSDMB^NT^	Malignant melanoma	[95]
GSDMB	IBI315	Bispecific antibody	‐	Gastric cancer	[96]
GSDMC	Lmo@RBC	CDT	caspase‐8	Colon cancer	[97]
GSDMD ^up‐regulated^	AOZN	CDT	caspase‐1	Malignant melanoma	[98]
GSDME^up‐regulated^	BNP	PDT	caspase‐3	Breast cancer	[99]
GSDME^up‐regulated^	TSD@LSN‐D	PDT	caspase‐3	Breast cancer	[100]
GSDME^up‐regulated^	ANP	PTT	caspase‐3	Breast cancer	[101]
GSDME^up‐regulated^	LipoDDP	Chemotherapy	caspase‐3	Breast cancer	[102]
GSDME^up‐regulated^	Nano‐CD	Chemotherapy	caspase‐3	Malignant melanoma	[103]
GSDME^up‐regulated^	DAC@HfO_2_	Radiotherapy	caspase‐3	Breast cancer	[104]
GSDME^up‐regulated^	PWE	Radiotherapy	caspase‐3	Breast cancer	[105]

Photothermal therapy (PTT), Photodynamic therapy (PDT), Chemodynamical therapy (CDT), Sonodynamic therapy (SDT), Engineered bacterial or viral therapy (BVT).

### 3.1 GSDMD‐Targeting Biomaterials for Pyroptosis and Antitumor Immunotherapy

GSDMD is a member of the protein family that is located on the cell membrane and is the principal executor of cell death in the noncanonical inflammasome signaling cascade, particularly in the context of inflammation and immune dysfunction.^[^ ^6^, ^106^ ^]^ As the pioneering executor of pyroptotic cell death, GSDMD is cleaved after the activation of intracellular inflammasomes, such as the NLRP3 inflammasome, as well as after stimulation by other mediators, such as IL‐1β.^[^ ^7^, ^19^, ^22^, ^107^ ^]^

In cancer therapy, researchers strategically use the pore‐forming mechanism of GSDMD to enhance the antitumor immune response. GSDMD can be specifically activated by employing stimuli such as light, chemicals, or acoustic signals for proteolytic cleavage and transmembrane insertion. This process leads to programmed cell death (usually pyroptosis) and the release of proinflammatory cytokines and chemokines. These molecules act as chemical attractants to attract immune cells (including cytotoxic T cells and macrophages) to the tumor site, thereby enhancing immune surveillance and the clearance of cancer cells.

#### 3.1.1 Activating the GSDMD Protein by Using Biomaterials Capable of PTT‐ or PDT‐Initiating

**Figure 3:**
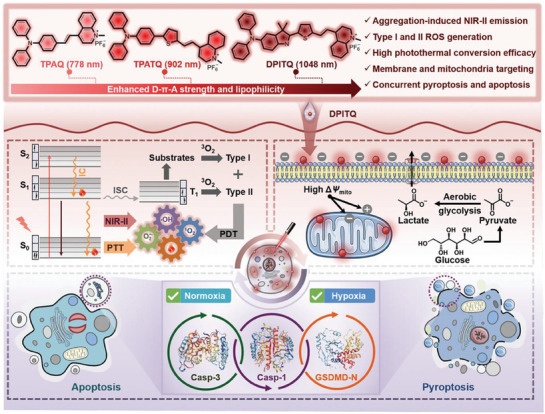
Schematic depicting the molecular design concept of NIR‐II PS DPITQ and its bioapplications. This study focuses on molecular engineering to manipulate electron donors, creating a novel aggregation‐induced NIR‐II emission photosensitizer (DPITQ). Under 635 nm laser irradiation, dual‐targeted hypoxic tumor therapy is achieved by inducing the synergistic effects of pyroptosis and apoptosis. Reproduced with permission.^[^ ^31^ ^]^ Copyright 2024, Wiley‐VCH.

PTT is a noninvasive or minimally invasive treatment method that uses electromagnetic radiation for the purpose of destroying target cells.^[^ ^108^ ^]^ To achieve this goal, a photothermal agent is delivered to the tumor area using a targeted delivery system.^[^ ^109^ ^]^ Once the photothermal agent accumulates to a sufficient level in the tumor site, infrared light is administered. When the photothermal agent absorbs light energy and converts it into heat energy, the temperature rapidly increases in the local environment surrounding the cancer cells, resulting in thermal ablation of the tumor tissue and tumor cell death. Processes that occur during cell death include protein denaturation, cell membrane rupture, and the induction of programmed cell death signaling cascades.^[^ ^110^ ^]^ Currently, a considerable amount of research is being conducted on photothermal agents with high photothermal conversion efficiency, particularly in relation to the treatment of tumors through PTT by inducing GSDMD protein cleavage to induce pyroptosis. Hu et al.^[^ ^30^ ^]^ synthesized VB12‐sericin‐PBLG‐IR780 nanomicelles by coupling p poly(γ‐benzyl‐L‐glutamate) (PBLG)‐modified sericin derivatives with the tumor‐targeting agent vitamin B12 (VB12) and IR‐780 iodide (IR780). Under near‐infrared conditions, VB12‐sericin‐PBLG‐IR780 significantly damaged mitochondria and activated caspase‐1, which in turn triggered the cleavage of GSDMD protein and induced pyroptosis. To improve the accuracy and efficacy of PTT‐induced pyroptosis, recent research has focused on enhancing the targeting ability of photothermal agent delivery systems to reduce damage to normal tissues. In another study by Tang et al.,^[^ ^31^ ^]^ an external NIR‐II light source was combined with a photosensitizer (DPITQ) for aggregation‐induced emission (AIE), which enabled the targeting of both the plasma membrane and mitochondria by regulating the electron transfer process that occurs when a molecule is in a dispersed state, thereby facilitating dual‐targeted hypoxic tumor therapy. The suitable biocompatibility and appropriate lipophilicity of DPITQ facilitated its binding to the plasma membrane and mitochondria of cancer cells under complex in vivo conditions. Under the action of a laser, the integrity of the plasma membrane and mitochondrial membrane was disrupted, and finally, caspase‐1 was activated, which induced the cleavage of GSDMD protein and pyroptosis. The present study proposes a therapeutic strategy that activates pyroptosis and mediates antitumor immunity through the induction of mitochondrial oxidation, thereby providing substantial guidance for tumor immunotherapy (**Figure** **3**).

PDT is a medical treatment that uses photosensitive drugs in conjunction with light at a specific wavelength to eliminate targeted cells.^[^ ^111^ ^]^ When the photosensitizer absorbs light, it becomes excited, transferring energy to surrounding oxygen molecules and converting them into highly active singlet oxygen or other reactive oxygen species (ROS).^[^ ^112^ ^]^ These ROS subsequently dismantle the cellular structure, particularly the cell membrane, mitochondria, and other organelles, resulting in cell death through either apoptosis or pyroptosis. PDT is currently widely employed to activate mitochondrial damage and pyroptosis mediated by GSDMD because of its high specificity and minimal harm to normal tissues. Liu et al.^[^ ^113^ ^]^ developed a series of membrane‐anchored photosensitizers (TBD‐R PSs) with AIE properties through the coupling of a 1,1,2,2‐tetraphenylethene‐benzo[c] [1,2,5] thiadiazole‐2‐(diphenyl‐ methylene) malononitrile (TBD) and benzene rings with cationic chains. Under laser irradiation, cytotoxic ROS were generated, which led to direct membrane damage and exceptional eradication of cancer cells. These membrane‐targeted PSs can induce pyroptosis during cancer cell ablation with minimal invasiveness and side effects. The strong membrane anchoring ability of TBD‐R PSs enables it to induce ROS in situ, directly damaging the membrane and consequently leading to cancer cell death. This demonstrates that the pyroptosis pathway can play a significant role in the photodynamic ablation of tumor cells, highlighting its potential as a cancer treatment. Moreover, Mao et al.^[^ ^34^ ^]^ designed two Pt(II) complexes (Pt1 and Pt2) as photoactivators for the cyclic GMP‐AMP synthase (cGAS) – stimulator of interferon genes (STING) (cGAS‐STING) pathway. Under light, Pt1 and Pt2 damaged the mitochondrial membrane, activated caspase‐1, and triggered GSDMD protein‐mediated pyroptosis. Moreover, they induced deoxyribonucleic acid (DNA) damage and disrupted the nuclear membrane, causing DNA fragments to activate the cGAS‐STING pathway. The activation of this pathway then led to the release of many cytokines and increases in DCs antigen presentation, T‐cell infiltration, and the percentage of CD8^+^ T cells, thereby promoting antitumor immunotherapy. Owing to the high metabolism and high proliferation of tumor cells, the tumor microenvironment is often hypoxic, with a low pH and high glutathione concentration, and these characteristics contribute to the limited therapeutic effect of PDT. This pioneering study effectively addresses the detrimental impact of the tumor microenvironment on treatment outcomes by inducing pyroptosis and concurrently activating the cGAS‐STING pathway. In addition, targeting the hypoxic tumor microenvironment, Mao et al.^[^ ^41^ ^]^ designed a carbonic anhydrase IX (CAIX)‐anchored rhenium (I) photosensitizer (CA‐Re), which can perform effective type‐I and type‐II PDT under hypoxic conditions, stimulate pyroptosis cell death mediated by GSDMD, and effectively enhance the immunogenicity of tumors. Moreover, to induce pyroptosis, Ge et al.^[^ ^47^ ^]^ designed a copper bacterial chlorine nanosheet (Cu‐TBB), that was activated in response to high glutathione (GSH) levels in the tumor microenvironment, leading to the release of Cu^+^ and bacteriochlorin (TBB). The released Cu^+^ triggered a cascade reaction, generating superoxide(O_2_^−•^) and highly toxic hydroxyl radical (•OH) in cells. Moreover, under 750 nm laser irradiation, the released TBB generated O_2_^−•^ and singlet oxygen(^1^O_2_). The cascades driven by Cu^+^ and pathways activated by PDT induced GSDMD‐mediated pyroptosis, resulting in dendritic cell maturation and robust T‐cell‐initiated immune responses and effectively eliminating primary tumors. Moreover, distant tumor growth and metastasis was inhibited. Surface Cu‐TBB nanosheets selectively induced pyroptosis both in vitro and in vivo, enhancing tumor immunogenicity and antitumor efficacy while minimizing systemic side effects. Despite the vast potential of metal‐based PSs in PDT, their limited tumor‐targeting ability restricts their application. Xia et al.^[^ ^40^ ^]^ developed a metal‐based PS‐bacterial mixture, E. coli Nissle 1917 labeled with iridium(III) photosensitizer (Ir‐HEcN), by attaching an iridium(III) PS onto the surface of genetically engineered bacteria. The tumor‐targeting capabilities of genetically engineered bacteria can be utilized to deliver PDT drugs directly to the tumor site, where they generate a substantial amount of ROS upon laser irradiation, thereby activating GSDMD‐mediated pyroptosis. Concurrently, combined immunotherapy may be employed for solid tumors that demonstrate limited responsiveness to conventional treatments, with the potential for achieving effective cures. At this point, several studies have reported that the combination of PDT with PTT can achieve effective pyroptosis induction. Li et al.^[^ ^42^ ^]^ employed a tumor cell membrane‐targeted luminescent photosensitive dimer with AIE properties, which achieved effective tumor immunotherapy through the synergistic effect of PDT and PTT. Photosensitive dimers can kill tumor cells through both PDT and PTT by inducing antitumor immune effects and establishing immune memory. The induction of pyroptosis has been demonstrated to promote the maturation of dendritic cells, initiate adaptive antitumor responses and generate significant immune memory effects. In this study, which employed a low‐immunogenic 4T1 tumor mouse model, pyroptosis‐mediated photothermal/photodynamic immunotherapy resulted in the complete elimination of the primary tumor by the seventh day of treatment, while effectively inhibiting the growth of distant tumors. This approach consequently reduces the probability of tumor recurrence and metastasis. Moreover, visible photosensitive dimers show considerable promise for use in synergistic photoimmunotherapy.

#### 3.1.2 Activating the GSDMD Protein by Using Biomaterials Capable of CDT‐Initiating

**Figure 4:**
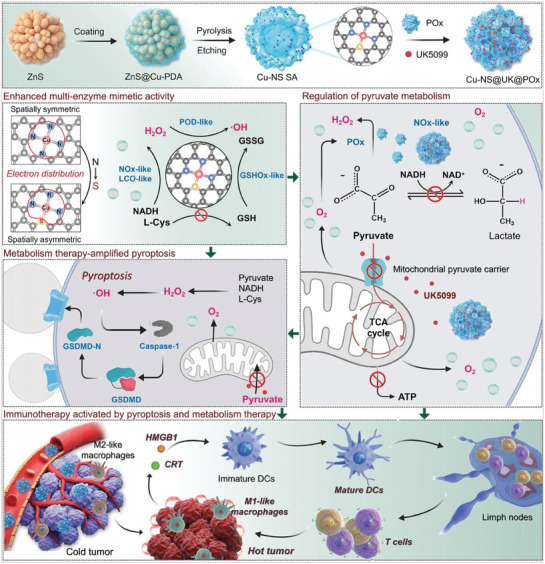
Illustrates both the synthesis of Cu‐NS@UK@POx and the mechanism by which the antitumor immune response is facilitated through Cu‐NS@UK@POx reprogramming cellular metabolism and triggering pyroptosis. The introduction of sulfur (S) enhances the asymmetric coordination environment active site's electron transfer capability, significantly boosting the multi‐enzyme activities of Cu‐NS SA. Additionally, the programmed targeted pyruvate metabolism therapy improves local O_2_/H_2_O_2_ levels while depleting lactic acid (LA) /ATP, providing enzyme substrates and improving the tumor immune microenvironment (TIME). Notably, this metabolic therapy can amplify pyroptosis activated by single‐atom nanozymes via a ROS‐dependent, caspase‐1‐related pathway. The combined effects of pyroptosis and metabolic regulation lead to a potent antitumor immunotherapy. Reproduced with permission.^[^ ^65^ ^]^ Copyright 2024, Wiley‐VCH.

CDT is a treatment strategy that uses Fenton or Fenton‐like reactions to produce •OH in the tumor region. Various systems that enable controlled and dynamic drug delivery, such as responsive drug delivery systems that release drugs in response to specific triggers, including pH, temperature, or enzymes found in the tumor microenvironment are used in CDT.^[^ ^114^ ^]^ CDT also involves the use of chemical reactions to specifically target cancer cells, such as prodrug activation reactions and chemical reactions that generate mechanical forces to destroy cancer cells and their surroundings.^[^ ^46^ ^]^ This therapy has gained substantial traction in targeted drug delivery, immunotherapy, and precision medicine because of its specific activation methods. Researchers have designed biomaterials that exploit the unique characteristics of the tumor microenvironment, such as low pH levels and high concentrations of hydrogen peroxide (H_2_O_2_) and GSH.^[^ ^115^ ^]^ These biomaterials trigger pyroptosis by damaging cancer cell mitochondria through the production of ROS or other peroxides. One example of CDT is the development of a novel single‐atom nanozyme pyroptosis initiator by Zhang et al.^[^ ^65^ ^]^ The initiator coloaded a Cu‐based single‐atom nanozyme with an asymmetric coordination environment (Cu‐N3S1), which was loaded with pyruvate oxidase (POx) and the mitochondrial pyruvate carrier (MPC) inhibitor UK5099 (Cu‐NS@UK@POx). It not only stimulates pyroptosis through biocatalysis‐mediated cascade reactions to increase the immunogenicity of tumor cells but also reshapes the immunosuppressive tumor microenvironment by targeting pyruvate metabolism. This dual therapeutic strategy has the potential to enhance cellular immunogenicity, induce dendritic cell maturation, and further activate T cell‐related immune responses. Furthermore, it facilitates the effective repolarization of the M2 phenotype to the M1 phenotype, thereby remodeling the immune microenvironment and significantly activating antitumor immunity. This study marks a pioneering application of single‐atom nanozymes in antitumor immunotherapy, whereby pyroptosis and metabolic therapy are triggered (**Figure** **4**). Another study by Li et al.^[^ ^59^ ^]^ focused on designing copper‐quinone‐glucose oxidase (Gox) nanoparticles (CQG NPs) with the ability to self‐destruct and multienzyme activity. These nanoparticles inhibit the nuclear transcription factor erythroid 2‐related factor 2 (NRF2)‐quinone oxidoreductase 1 (NQO1) signaling pathway, thereby compromising the antioxidant defence mechanism of tumor cells. Additionally, the nanoparticles activate NLRP3‐mediated pyroptosis through their exceptional multienzyme activity. The release of Cu^2+^ ions by the self‐destruction of CQG NPs triggers cuproptosis, whereas the depletion of endogenous copper chelators through the Michael addition reaction between GSH and quinone ligands, the production of oxygen via a catalase‐like reaction, and starvation‐induced glucose deficiency increase the sensitivity of cancer cells to cuproptosis. Both CQG NPs induced pyroptosis and cuproptosis facilitate remodeling of the immunosuppressive tumor microenvironment, increase immune cell infiltration into tumors, and activate a robust systemic immune response.

In addition to the generation of numerous peroxides that harm mitochondria, the increase in osmotic pressure resulting from the accumulation of high levels of cations in cells can stimulate caspase‐1 to initiate GSDMD protein‐mediated pyroptosis. Engelke et al.^[^ ^50^ ^]^ developed lipid‐coated iron‐based metal‒organic framework nanoparticles. When the lipid coating is utilized to promote endocytosis, the nanoparticles are subsequently degraded in lysosomes through cysteine‐mediated reduction. This process releases a significant quantity of iron ions into cells within a mildly acidic extracellular environment, causing alterations in the intracellular and extracellular osmotic pressures and ultimately leading to pyroptosis. The resulting pyroptosis has a direct effect on primary tumors and induces pyroptosis‐mediated antitumor immune responses. Researchers are increasingly integrating strategies involving the generation of ROS and cations to effectively induce pyroptosis. Zhang et al.^[^ ^58^ ^]^ designed a sulfate radical donor (Na_2_S_2_O_8_) and encapsulated it in liquid nanoparticles. By modifying the pH‐responsive tannic acid‐Fe^2+^ framework on the surface of liquid nanoparticles, these liquid nanoparticles were able to be released in the tumor microenvironment after reaching the tumor in response to the low pH and high GSH levels of the tumor microenvironment, where they produced large amounts of ROS and Na^+^. The presence of Na⁺ and ROS storms results in an increase in intracellular osmotic pressure, which subsequently promotes the pyroptosis of cancer cells. This synergistic effect has the potential to enhance therapeutic outcomes. The synthesis of engineered liposomes that respond to the tumor microenvironment has significant implications for the design of nanocarriers intended for future “ionic drug” therapeutic applications. This process effectively triggers GSDMD protein‐mediated pyroptosis, which has important implications for the development of novel therapeutic strategies. Lin et al.^[^ ^57^ ^]^ developed basic sodium bicarbonate nanoparticles (NaHCO_3_ NPs) via a rapid microemulsion method to enhance the efficacy of cancer immunotherapy. Weakly alkaline NaHCO_3_ regulates the acidic tumor microenvironment via acid‒base neutralization. In addition, it releases large amounts of Na^+^ ions in tumor cells, increasing the intracellular osmotic pressure and activating the pyroptosis pathway and immunogenic cell death (ICD).

#### 3.1.3 Activating the GSDMD Protein by Using Biomaterials Capable of SDT‐Initiating

**Figure 5:**
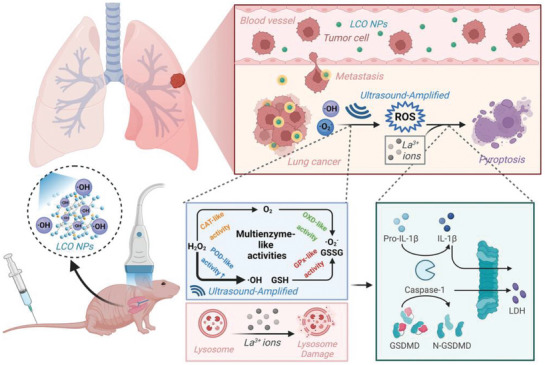
Schematic diagram of LaCoO_3_ nanocrystals with multi‐enzymatic properties that trigger the generation of ROS and the release of La^3+^ under ultrasound, ultimately leading to the pyroptosis of lung cancer cells. In this study, LCO lanthanide nanocrystals with multi‐enzyme properties were intelligently designed and modified to induce the production of cytotoxic ROS and the release of lanthanum ions, thereby triggering pyroptosis of lung cancer cells and inhibiting tumor growth and metastasis. Reproduced with permission.^[^ ^66^ ^]^ Copyright 2023, Wiley‐VCH.

SDT is a noninvasive medical treatment that combines ultrasound and PSs for the treatment of various diseases, particularly cancer.^[^ ^116^ ^]^ SDT, although similar to PDT, uses ultrasonic energy instead of light energy.^[^ ^117^ ^]^ Due to its excellent selectivity, low systemic toxicity, and potential for deep tissue penetration, SDT has gained increasing attention. Numerous studies have employed SDT to activate the GSDMD protein and induce pyroptosis. For example, Yao et al.^[^ ^66^ ^]^ developed lanthanide nanocrystals of LaCoO_3_ (LCO) with multienzyme properties, initiating the production of cytotoxic ROS and the release of lanthanum ions, ultimately inducing pyroptosis in lung cancer cells. The peroxidase activity of LCO nanocrystals enables them to produce ROS in an acidic and highly hydrogen peroxide‐containing tumor microenvironment. In addition, LCO nanozymes can also exhibit catalase and glutathione peroxidase‐like activities, effectively alleviating hypoxia in the tumor microenvironment and increasing the sensitivity of tumor cells to ROS. More importantly, LCO destroys the lysosomal membrane through the release of La^3+^ ions, eventually leading to typical pyroptosis. In vivo experiments in mice revealed that nanocrystal‐induced programmed pyroptosis increased the therapeutic effect in vitro and in vivo and effectively inhibited the growth and metastasis of lung cancer. This study presents a novel approach based on nanomedicine with the objective of inducing pyroptosis in tumor cells and inhibiting the metastasis of cancer (**Figure** **5**). Chen et al.^[^ ^67^ ^]^ synthesized LaFeO_3_ perovskite nanocrystals, which mimicked the activity of four enzymes (including oxidase, peroxidase, glutathione peroxidase, and catalase), alleviated the hypoxic microenvironment, depleted endogenous glutathione, and continuously released ROS. Moreover, exogenous ultrasound stimulation enhanced the conversion rate of the intermediate complex to Fe(II), facilitating increased ROS production. The overproduction of ROS triggers the ROS‐ thioredoxin‐interacting protein (TXNIP)‐NLRP3‐GSDMD pyroptosis pathway. Both in vitro and in vivo experiments have verified the ability of LaFeO_3_ nanozymes to induce pyroptosis and display antitumor effects.

#### 3.1.4 Activating the GSDMD Protein by Using Biomaterials Capable of BVT‐Initiating

**Figure 6:**
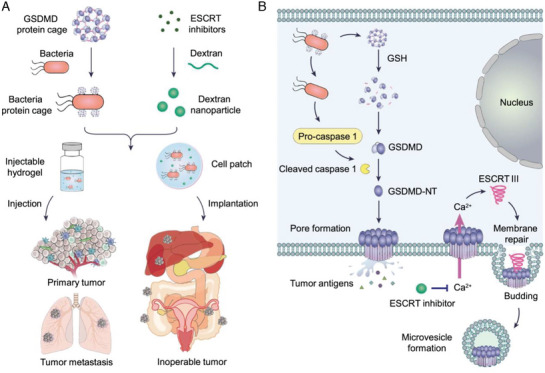
The preparation process of hydrogel‐based bacterial protein cage delivery system and its mechanism map of inducing pyroptosis. A) The GSDMD protein was cross‐linked into a protein cage and then coupled to the surface of VNP‐GD. At the same time, ESCRT inhibitors were loaded in EI‐NP. The two are mixed to prepare injectable hydrogels and cell patches to treat tumors by in situ implantation. B) VNP‐GD triggers tumor pyroptosis by activating GSDMD, and EI‐NP inhibits membrane repair to promote the potential mechanism of tumor cell pyroptosis efficiency. Reproduced with permission.^[^ ^69^ ^]^ Copyright 2022, Springer Nature.

Bacteria and viruses can be designed to target tumor cells, inhibit their growth, and activate the host immune system antitumor response.^[^ ^57^ ^]^ This type of therapy, known as microbial‐mediated cancer therapy or biotherapy, involves genetically modifying bacteria such as *Salmonella* and *Escherichia coli* to specifically colonize tumor tissue and kill cancer cells through various mechanisms.^[^ ^118^ ^]^ Current research has focused on using bacteria or viruses as anticancer drugs or gene therapy vectors or harnessing their inflammatory characteristics to induce absent in melanoma 2 (AIM2) inflammasome formation and GSDMD protein‐mediated pyroptosis. For example, Hu et al.^[^ ^69^ ^]^ created a biodegradable calcium chelator nanoparticle (EI‐NP) that achieved sustained release, which inhibited endosomal sorting complexes required for transport (ESCRT) iii‐dependent membrane repair initiated by calcium influx. Moreover, the researchers utilized a bacterial‐based system known as VNP‐GD to improve the targeted delivery of cellular substances and activate pyroptosis induced by the GSDMD protein. Additionally, they engineered injectable and freeze‐dried hydrogels for peritumoral injection and implantation, respectively, aimed at addressing both primary tumors and metastatic or inoperable tumors. These hydrogels consistently released VNP‐GD, which effectively induced tumor pyroptosis, whereas the gradual release of EI‐NP hampered the repair of the plasma membrane and intensified pyroptosis. This treatment strategy may be further enhanced by the addition of immune checkpoint blockade (ICB), thereby improving the efficacy of immunotherapy for primary and metastatic tumors, as well as for those that are difficult to operate on. Furthermore, researchers have put forth the hypothesis of combining pyroptosis with adoptive T‐cell therapy and cancer vaccines (**Figure** **6**). Similarly, Qu et al.^[^ ^70^ ^]^ created an adaptive inducer of pyroptosis (LPZ) by fusing *Lactobacillus rhamnosus GG* (LGG) with an enzyme‐mimicking metal‒organic framework (MOF) to enable effective immunotherapy by targeting pyroptosis. LPZ bound to the membranes of cancer cells through interactions between LGG pili and mucin, leading to the formation of an acidic microenvironment that increased ROS production via nanozymes. Upon binding to the cancer cell membrane, LPZ stimulates the production of lactic acid through the anaerobic respiration of LGG. This process gradually creates an optimal catalytic microenvironment for LPZ on the cell surface. Concurrently, bursts of reactive oxygen species (ROS) are generated within the cancer cell membrane and the cells themselves, which then trigger pyroptosis. It is noteworthy that LGG is ultimately eliminated by ROS during this process, which serves to mitigate potential biosafety concerns. Moreover, the combination of LPZ with 2,3‐dioxygenase (IDO) inhibitors has been observed to significantly enhance immune activation and effectively suppress tumor growth. This presents a promising strategy for the development of adaptive nanocatalytic drugs with the potential to suppress tumor growth, inhibit tumor metastasis and recurrence. Additionally, Tan et al.^[^ ^71^ ^]^ developed a self‐assembled virus‐like particle that consisted of an elongated DNA sequence produced through rolling circle amplification (RCA) that was enveloped in cationic liposomes. This particle facilitated the liquid phase condensation of cGAS, thereby activating the STING pathway to generate inflammatory cytokines. Additionally, it initiates the formation of the AIM2 inflammasome, mediates pyroptosis through the GSDMD protein, and enhances antitumor immune responses. The findings of this study highlight the clinical potential of cancer immunotherapy with RCA‐derived products due to their natural immunogenic properties, supporting their use in biomedical applications.

In summary, current strategies for activating GSDMD‐mediated pyroptosis involve two main approaches. The first involves generating a substantial amount of peroxide within the cell, leading to oxidative stress that damages both mitochondrial and cell membranes. This process activates NLRP3 and subsequently cleaves the GSDMD protein, thereby inducing pyroptosis. The second approach involves engineering bacteria and their derivatives to directly induce the recognition of PAMPs in cells, which leads to GSDMD cleavage and triggers pyroptosis. However, both activation strategies have notable shortcomings. Specifically, the generation of excessive peroxide may result in crosstalk with cellular ferroptosis, potentially leading to a reduced release of inflammatory cytokines. Moreover, the use of engineered bacteria and their derivatives to directly induce pyroptosis raises important safety concerns.

### 3.2 GSDME‐Targeting Biomaterials for Pyroptosis and Antitumor Immunotherapy

A recent study demonstrated that GSDME can be cleaved by caspase‐3 at a similar cleavage site as that cleaved by caspase‐1. This cleavage leads to the formation and release of an N‐terminal domain that possesses pore‐forming activity, thereby inducing pyroptosis. Importantly, caspase‐3 was initially identified as an enzyme associated with apoptosis.^[^ ^119^ ^]^ While chemotherapy drugs are known to trigger caspase‐3‐mediated apoptosis to eliminate cells, Shao et al. discovered that in SH‐SY5Y and B16F10 cells expressing high levels of GSDME, and that treatment with various chemotherapy drugs, including the DNA‐binding/modifying agents doxorubicin, cisplatin, and actinomycin‐D, as well as the topoisomerase inhibitors topotecan, camptothecin (CPT‐11), etoposide, and mitoxantrone, could induce pyroptosis.^[^ ^21a^ ^]^ On the other hand, GSDME‐negative Jurkat cells underwent apoptosis in response to doxorubicin, topotecan, etoposide, and actinomycin‐D treatment. Notably, this study revealed that pyroptosis occurred at a higher rate than apoptosis in cells with GSDME protein overexpression, highlighting the prioritized role of pyroptosis in this context.

Several subsequent studies have consistently demonstrated that increased expression of the GSDME protein in cells can result in its cleavage upon exposure to conventional apoptosis‐inducing treatments, such as chemotherapy and radiotherapy, thereby triggering pyroptosis.^[^ ^21a^ ^]^ However, given the intense inflammatory response associated with pyroptosis, a pressing research challenge in is effectively enhancing the specificity of these therapies and mitigating damage to normal tissues, which may culminate in severe cytokine storm' reactions.

#### 3.2.1 Activating the GSDME Protein by Using Biomaterials Capable of Chemotherapy‐Initiating

Chemotherapy is a fundamental medical intervention in cancer treatment that involves the administration of specific drugs. These drugs, which are commonly classified as anticancer or chemotherapy drugs, function by disrupting the growth and division of cancer cells, effectively eliminating actively multiplying cancer cells or impeding their metastasis. The mechanisms through which chemotherapy operates are diverse and encompass various strategies, such as affecting DNA replication, hindering cell mitosis, and inhibiting tumor angiogenesis, among others.

**Figure 7:**
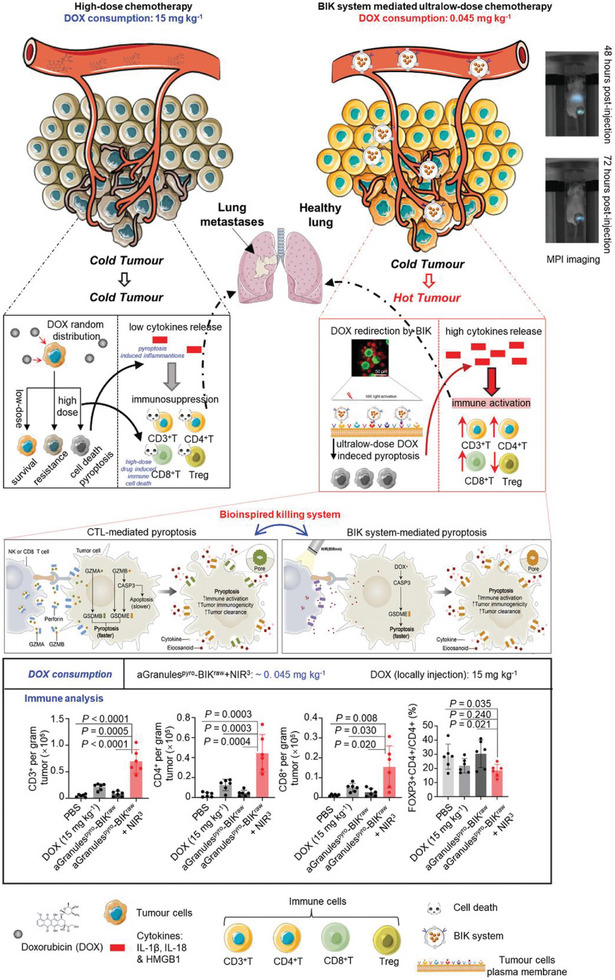
Schematic illustrations of the significant differences in pyroptosis‐induced tumor immune response by high‐dose chemotherapy versus BIK system‐mediated ultra‐low‐dose chemotherapy. This study simulated CTL‐mediated cell death, enabling the BIK system to achieve tumor targeting without inflammatory risk and systematically demonstrated that ultra‐micro amounts of DOX (less than 25 µg kg^−1^) are sufficient to cause tumor regression and induce pyroptosis‐type antitumor immune response. Reproduced with permission.^[^ ^78^ ^]^ Copyright 2023, Wiley‐VCH.

Chemotherapy has been shown to induce GSDME‐mediated pyroptosis by activating caspase‐3. However, the poor targeting ability and drug resistance of chemotherapeutic drugs limit their efficacy. To address these limitations, several studies are currently exploring nanosystems to enhance chemotherapy‐induced pyroptosis in tumor treatment. Wang et al.^[^ ^78^ ^]^ developed an innovative system utilizing gold nanorod‐engineered macrophages, which were processed via liquid nitrogen freezing, to create a bioinspired killing (BIK) system controllable by near‐infrared light. This system triggered pyroptosis via ultralow‐dose chemotherapy, leading to robust antitumor immune responses. The BIK system reduced the required drug dose to under one‐thirtieth of the standard dose in vivo and demonstrated efficient tumor targeting and penetration in vivo. Research indicates that even at minimal doses of doxorubicin (DOX), this system can trigger pyroptosis and successfully reduce tumor size without harming the primary organs. Notably, the combination of ultralow doses of DOX and anti‐programmed death ligand (αPD‐1) antibodies markedly suppressed the progression of advanced large‐volume EMT6 tumors. This study is the first to compare the effects of low‐dose and high‐dose chemotherapy on the induction of pyroptosis‐mediated anti‐tumor immune function. The findings suggest that low‐dose chemotherapy is more effective in triggering this immune response (**Figure** **7**). Yu et al.^[^ ^79^ ^]^ formulated an amphiphilic polymer (PHDT‐Pt‐In) that incorporates indomethacin, a cyclooxygenase‐2 (COX‐2) inhibitor, along with a platinum prodrug (Pt(IV)), which is responsive to GSH. This polymer organized itself into nanoparticles (Pt‐In NPs), which broke down within cancer cells in response to high GSH levels, thereby releasing indomethacin to suppress COX‐2 production. This novel strategy overcame resistance to chemotherapy and enhanced the degree of pyroptosis induced by cisplatin. In a pancreatic cancer mouse model, Pt‐In NPs significantly hindered tumor growth while eliciting both innate and adaptive immune responses. Furthermore, in conjunction with the anti‐programmed death ligand (αPD‐L1), Pt‐In NPs entirely eradicated metastatic tumors, converting “cold tumors” into “hot tumors.”

#### 3.2.2 Activating the GSDME Protein by Using Biomaterials Capable of PDT‐or PTT‐Initiating

**Figure 8:**
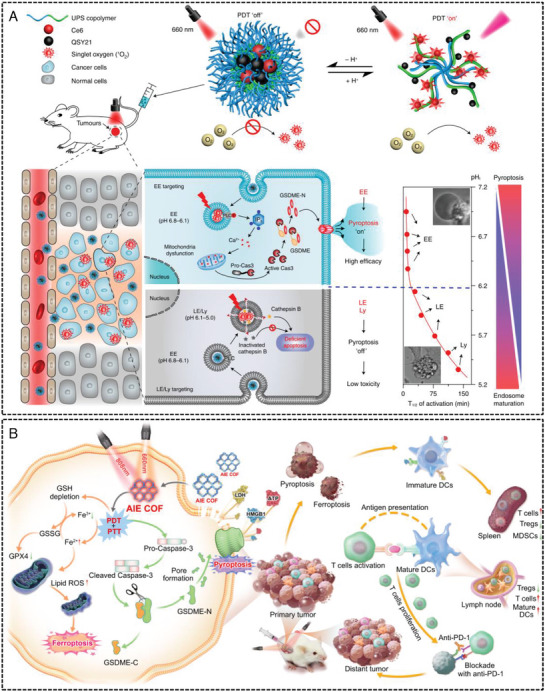
PTT and PDT therapy activate GSDME protein to induce tumor cell pyroptosis strategy. A) Schematic illustrations of ANPS and the tunable pyroptosis evoked by the ANPS library through the temporal differentiation of endosome maturation. Reproduced with permission.^[^ ^10^ ^]^ Copyright 2022, Springer Nature. B) Schematic diagram of COF‐919 generating ROS and iron ions under light control to induce pyroptosis for tumor immunotherapy. Reproduced with permission.^[^ ^81^ ^]^ Copyright 2023, Springer Nature.

PDT activates PSs to generate cytotoxic ROS and induces pyroptosis by damaging mitochondria and activating caspase‐3 to cleave GSDME. In the context of tumor immunotherapy, numerous studies have investigated PDT‐induced GSDME‐mediated pyroptosis. Wang et al.^[^ ^10^ ^]^ created a library of acid‐activated nanophotosensitizers (ANPs) that facilitate the modulation of pyroptosis via targeting of mature endosomes. Specifically, pyroptosis mediated by GSDME was activated in early endosomes through phospholipase C signal transduction, whereas pyroptosis decreased when the acid‐activated nanophotosensitizers were moved to late endosomes or lysosomes. This ANP categorises the endolysosomal pathway into ten distinct maturation stages, characterised by a pH interval of 0.2 in both spatial and temporal dimensions. ANPs serve as effective tools for introducing photodynamic oxidative stress into specific acidic endocytic regions, thereby facilitating location‐dependent and organelle‐specific modulation of pyroptotic cells. The study demonstrated significant efficacy in the killing of various GSDME‐positive cancer cells while simultaneously minimising systemic toxicity. This offers new avenues for the rational design of nanomedicines with pyroptosis‐regulating activity for tumor immunotherapy (**Figure** **8**A). Xiao et al.^[^ ^87^ ^]^ developed a tumor‐targeted nanodiagnostic and treatment system that integrated PDT with epigenetic treatment to simultaneously induce pyroptosis and activate the cGAS‐STING pathway in a light‐responsive manner. In this method, reactive oxygen nanoparticles (designated NP1), which carried the photosensitizer TBE, and decitabine‐loaded liposomes (referred to as NP2) were created. NP2 was responsible for restoring STING and GSDME expression, while PDT facilitated by NP1 increased the release of DNA fragments from compromised mitochondria, thus increasing the activity of the cGAS‐STING pathway and assisting in the activation of calpain I. This process resulted in the cleavage of GSDME, which was upregulated, into the GSDME‐N‐terminus, leading to pore formation. The released inflammatory cytokines subsequently enhanced the maturation of antigen‐presenting cells and initiated T‐cell‐mediated immune responses against the tumor. This research introduced a targeted approach for the concurrent photoactivation of pyroptosis and the cGAS‐STING pathway, promoting targeted photoimmunotherapy for immune‐tolerant tumors. Peng et al.^[^ ^88^ ^]^ created a novel chimeric nanostructure (L@NBMZ) that integrates bromodomain‐containing protein 4 (BRD4) – Proteolysis targeting chimera (PROTAC) with PSs to serve as an enhancer for light‐induced pyroptosis in breast cancer cells. L@NBMZ was able to successfully degrade BRD4 and exhibited robust biosafety and biocompatibility. It effectively blocked gene transcription via the proteasomal degradation of BRD4 in vivo and unexpectedly triggered the cleavage of caspase‐3. This cleavage of caspase‐3 was further enhanced by light exposure in the presence of PSs, leading to successful light‐mediated pyroptosis. This nanoenhancer has been observed to activate innate immunity in vivo when exposed to light irradiation, and has demonstrated the ability to inhibit tumor regeneration and metastasis. It is anticipated that the PROTAC enhancer assembly strategy presented in this study will make a significant contribution to the current and future antitumor paradigms, thereby expanding the applications of PROTAC and providing a valuable reference for the development of light‐driven PROTAC.

PTT is a method that uses photothermal agents to convert light energy into heat energy, which can then be used to induce thermal ablation of tumor tissue and activate caspase‐3 to induce pyroptosis by cleaving the GSDME protein. Currently, numerous studies have focused on the precise and controllable induction of GSDME‐mediated pyroptosis through the design of targeted nanophotothermal agents and related delivery systems. In addition, several recent studies have combined PTT with PDT to increase the killing effect of PDT through the photothermal effect of PTT, resulting in more efficient induction of pyroptosis than single‐treatment approaches. For example, Sun et al.^[^ ^81^ ^]^ developed a covalent organic framework (COF‐919) that serves as a dual inducer of pyroptosis and ferroptosis, thereby enhancing antitumor immunity. Research indicates that COF‐919 can successfully absorb near‐infrared light with a low energy bandgap and a long half‐life, all of which facilitate the generation of ROS and promote photothermal conversion, ultimately resulting in pyroptosis. The ability of a compound to trigger ROS production leads to increased intracellular lipid peroxidation, resulting in depleted glutathione levels, reduced expression of glutathione peroxidase 4, and a decline in iron levels. As a result, COF‐919 effectively induces both pyroptosis and ferroptosis, significantly preventing tumor metastasis and recurrence, achieving a tumor growth inhibition rate exceeding 90% and a cure rate surpassing 80%. The induction of pyroptosis and ferroptosis by this material has been demonstrated to enhance T cell‐mediated immune responses, thereby facilitating the inhibition of tumor metastasis and recurrence. This study presents a novel strategy for the design of dual inducers of pyroptosis and ferroptosis, offering a novel perspective on tumor immunotherapy (Figure 8B). Similarly, Sun et al.^[^ ^80^ ^]^ developed a series of multienzyme‐simulated covalent organic frameworks (COFs), such as COF‐909‐Cu, COF‐909‐Fe, and COF‐909‐Ni, which act as inducers of pyroptosis and thereby alter the tumor microenvironment, advancing research in cancer immunotherapy. Mechanistic investigations have revealed that these COFs disrupt the homeostasis of H_2_O_2_, resulting in elevated intracellular levels of H_2_O_2_. In addition, they demonstrate excellent activity as superoxide dismutase (SOD) mimetics, transforming O_2_^−•^ into H_2_O_2_ and facilitating its generation. Moreover, these COFs possess outstanding glutathione peroxidase (GPx) mimetic activity, utilizing GSH and decreasing the scavenging of H_2_O_2_. The remarkable photothermal therapeutic characteristics of these COFs can accelerate an ionization process akin to Fenton's reaction, thus improving the efficacy of CDT. A specific variant, COF‐909‐Cu, significantly induced GSDME‐dependent pyroptosis, remodelled the tumor microenvironment, initiated sustained antitumor immunity, enhanced the response rate to αPD‐1 checkpoint blockade, and effectively suppressed tumor metastasis and recurrence. Furthermore, Tang et al.^[^ ^82^ ^]^ reported that binding luminescent precursors exhibiting AIE characteristics (AIEgens) to the covalent organic frameworks (COF) framework via vinyl linkages serves as an effective method for creating nonmetallic pyroptosis inducers that increase antitumor immunity. Mechanistic investigations indicated that these COF systems not only enhance precursor luminescence but also exhibit a robust light absorption ability, a prolonged half‐life, and an elevated quantum yield, all of which are advantageous for producing ROS that trigger pyroptosis. Furthermore, in a bilateral 4T1 tumor model, the synergistic system of AIE‐COF and αPD1 effectively eliminates both primary and metastatic tumors while preventing tumor recurrence. The findings of this study offer valuable insights into the design of non‐metal pyroptosis inducers, which is of great significance for pyroptosis‐induced antitumor immunotherapy.

#### 3.2.3 Activating the GSDME Protein by Using Biomaterials Capable of CDT‐Initiating

**Figure 9:**
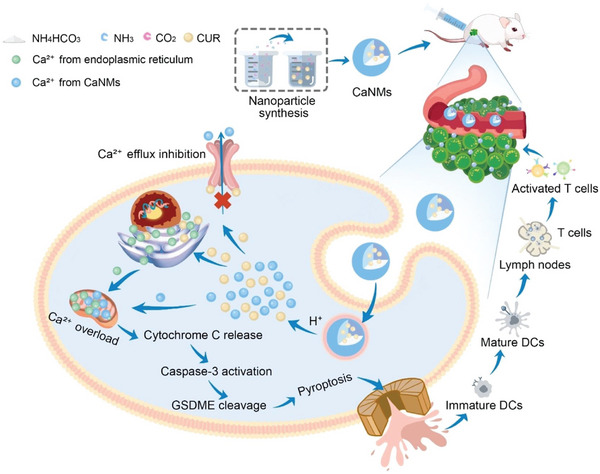
Synthesis of biodegradable Ca^2+^ nano‐modulator (CaNMs) and activation of pyroptosis via mitochondrial Ca^2+^ overload. CaNMs decompose and release Ca^2+^ and curcumin in low pH environments, causing a sudden influx of mitochondrial Ca^2+^ ions. This ultimately triggers pyroptosis, leading to antitumor immunity. Reproduced with permission.^[^ ^89^ ^]^ Copyright 2022, Wiley‐VCH.

Researchers have designed chemodynamic nanomaterials that respond to the low pH of tumor sites, as well as the high concentrations of H_2_O_2_ and GSH, to induce damage to mitochondria. This is accomplished through the production of ROS or by increasing intracellular cation levels. caspase‐3 activation leads to the cleavage of the GSDME protein and mediates pyroptosis. Hu et al.^[^ ^90^ ^]^ created chimeric nanomaterials (DeFer‐2) using PROTACs to trigger iron overload stress in cancer cells and investigated their subsequent behavior. They linked oleic acid, attached to the ferritin dimer, to the ligand of the Hippel‒Lindau (VHL) E3 ligase through an alkyl linker, resulting in the degradation of ferritin by the chimeric DeFer‐2 and a rapid increase in free iron levels. This process initiated caspase‐3 activation, leading to GSDME‐mediated pyroptosis in cancer cells, which was distinct from the usual ferroptosis linked with excess iron. The administration of DeFer‐2 in albumin‐based nanoformulations markedly reduced tumor growth and prolonged the life span of mice bearing B16F10 tumors. The study demonstrates that the degradation of ferritin results in an increase in intracellular free iron ions, which consequently leads to a rapid elevation in the intracellular free iron content. This process results in the generation of a substantial amount of reactive oxygen species (ROS) within the mitochondria, which ultimately induces pyroptosis. Moreover, the utilization of PROTAC‐based nanomedicines for pyroptosis has considerable implications for the advancement of antitumor therapies. Lin et al.^[^ ^89^ ^]^ developed biodegradable Ca^2+^ nanomodulators (CaNMs) designed as inducers of pyroptosis for cancer immunotherapy by promoting an overload of mitochondrial Ca^2+^. Degraded CaNMs released both Ca^2+^ and curcumin in low‐pH environments, leading to a rapid increase in mitochondrial Ca^2+^ levels, which ultimately induced pyroptosis. This research is the first to confirm the induction of pyroptosis due to mitochondrial Ca^2+^ overload and highlights the robust immune response elicited by CaNMs, which effectively slowed tumor growth and metastasis to the lungs. These results provide novel approaches and insights for cancer treatments that utilize pyroptosis and further facilitate the advancement of Ca^2+^ nanomodulator technology (**Figure** **9**).

#### 3.2.4 Activating the GSDME Protein by Using Biomaterials Capable of BVT‐Initiating

**Figure 10:**
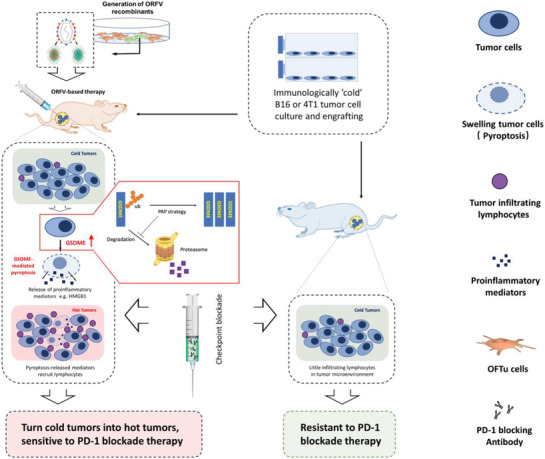
Schematic diagram of ORFV synthesis and induction of gsdme‐mediated pyroptosis. Both wild‐type ORFV and ORFV recombinants can induce GSDME‐mediated pyroptosis, thereby triggering antitumor immunity. This effect is observed in tumor cell lines, in vivo tumor tissues, and in vitro human colon cancer tissues. Reproduced with permission.^[^ ^92^ ^]^ Copyright 2023, Springer Nature.

The advantage of oncolytic viruses in tumor therapy lies in their ability to both directly kill tumors and promote antitumor immune responses. Oncolytic viruses are known to induce apoptosis, and recent studies have revealed that they can also induce pyroptosis in tumor cells expressing the GSDME protein. Several studies have demonstrated that oncolytic viruses can induce pyroptosis in GSDME‐positive tumor cells, thereby initiating antitumor immunity. For example, He et al.^[^ ^92^ ^]^ demonstrated that oncolytic parapoxvirus ovis (ORFV), along with its therapeutic recombinant variants, can induce pyroptosis in tumor cells via GSDME. This phenomenon is particularly pronounced in tumor cells with low GSDME expression. ORFV infection stabilized GSDME by decreasing its level of ubiquitination, which in turn triggered pyroptosis. Furthermore, the silencing of GSDME led to a decreased presence of cytotoxic T lymphocytes within the tumor, diminished pyroptosis, and a reduction in the efficacy of ORFV treatment against tumors. In experiments conducted in vivo, oncolytic viruses, when administered systemically, tended to preferentially localize to tumors and facilitate tumor destruction through pyroptosis. The findings of this study demonstrate that the ORFV strategy alters the tumor microenvironment, transforming “cold” tumors into “hot” tumors and rendering tumors that are resistant to αPD‐1 therapy susceptible to treatment. The ORFV strategy represents a promising approach to antitumor biological therapy, whether employed as monotherapy or in combination with checkpoint blockade (**Figure** **10**). In another study, Sun et al.^[^ ^93^ ^]^ described a therapeutic approach that integrates a dual‐responsive signal transducer targeting ROS and pH, alongside a Signal transducer and activator of transcription 3 (STAT3) inhibitor nanoprodrug (MPNP), with oncolytic herpes simplex virus 1 (HSV‐1) therapy to promote pyroptosis and increase the efficacy of immunotherapy. The MPNP effectively accumulated in tumors, diminished the stemness characteristics of tumor cells, and enhanced the antitumor immune response. Furthermore, the simultaneous application of oncolytic viruses increased the tumor penetration ability of MPNP, resulting in pronounced GSDME‐mediated pyroptosis, thus converting “cold” tumors to “hot” tumors and altering the tumor microenvironment. This study presents a novel approach to overcoming ICB resistance by effectively inducing pyroptosis. This process enhances tumor immunogenicity, initiates adaptive immune responses and generates robust T cell‐dependent antitumor immune memory effects, which are crucial for combating tumor recurrence and lung metastasis. This promising strategy may facilitate the development of personalised tumor treatments.

In summary, numerous strategies currently exist to mediate pyroptosis through the activation of GSDME. Given that GSDME can be cleaved by activated caspase‐3, traditional methods of inducing apoptosis, which leads to caspase‐3 activation, can result in pyroptosis rather than apoptosis when the GSDME protein is present, demonstrating great potential for the application of pyroptosis in tumor treatment. However, since most tumor cells either do not express or express low levels of the GSDME protein, increasing the GSDME protein content in target tumor cells has emerged as a critical issue in GSDME‐mediated pyroptosis strategies.

### 3.3 GSDMA‐Targeting Biomaterials for Pyroptosis and Antitumor Immunotherapy

**Figure 11:**
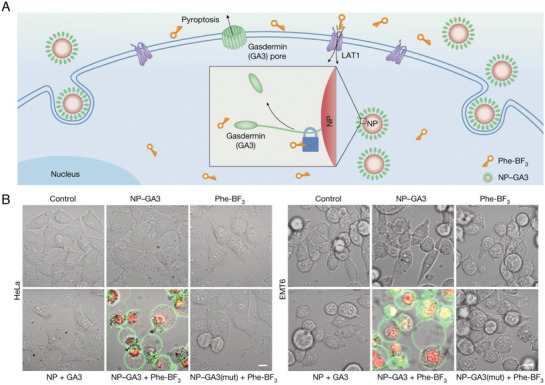
Schematic diagram of Phe‐BF_3_ demethylsilyl releasing gasdermin from NP‐GSDMA3 to induce pyroptosis. A) Mechanism map of pyroptosis induced by NP‐GSDMA3. B) Confocal images of the treated HeLa and EMT6 cells. Scale bars, 20 µm. Reproduced with permission.^[^ ^8a^ ^]^ Copyright 2020, Springer Nature.

The cleavage of GSDMA is triggered by PRRs that sense a series of activated signal transduction events, leading to the activation of inflammatory caspases (e.g., caspase‐1, ‐4, ‐5, or 11) in the caspase family. However, the GSDMA protein is predominantly expressed in immune cells (e.g., macrophages, neutrophils, and dendritic cells) and epithelial cells (e.g., the skin, intestines, and respiratory tract) and is only minimally expressed in tumor cells, limiting targeted GSDMA treatments. Interestingly, studies have demonstrated that introducing the GSDMA protein into tumor cells induces GSDMA‐mediated pyroptosis. Liu et al.^[^ ^8a^ ^]^ developed an innovative bioorthogonal chemical system utilizing the cancer imaging probe phenylalanine trifluoroborate (Phe‐BF_3_). This system is particularly noteworthy for its ability to “cleave” specifically designed linkers that contain silicon ether bonds. The integration of this chemical system with nanoparticle‐mediated delivery enhances its effectiveness, as the desilylation reaction induced by Phe‐BF_3_ facilitates the selective release of client proteins. Among these proteins is the active form of GSDMA, which was able to be directly delivered to tumor cells in a mouse model, highlighting the potential of this method in cancer treatment. The findings from this bioorthogonal system indicate that inducing pyroptosis—a form of programmed cell death—in less than 15% of tumor cells can trigger a robust antitumor immune response. This observation is important, as it suggests that even minimal targeted induction of pyroptosis in a selective subgroup of tumor cells can elicit a broad systemic antitumor immune response. The direct activation of gasdermin through Phe‐BF3‐mediated demethylation represents a significant mechanism for elucidating the antitumor immunity elicited by ICD. This finding also represents the inaugural demonstration of the antitumor effects associated with pyroptosis and its underlying mechanisms (**Figure** **11**).

The GSDMA protein is predominantly expressed in immune and epithelial cells, with minimal expression observed in tumor cells. Consequently, strategies for inducing GSDMA protein‐mediated pyroptosis are currently limited. A considerable challenge in recent research is generating strategies to effectively upregulate the GSDMA protein in tumor cells while ensuring precise targeting of its activation.

### 3.4 GSDMB‐Targeting Biomaterials for Pyroptosis and Antitumor Immunotherapy

**Figure 12:**
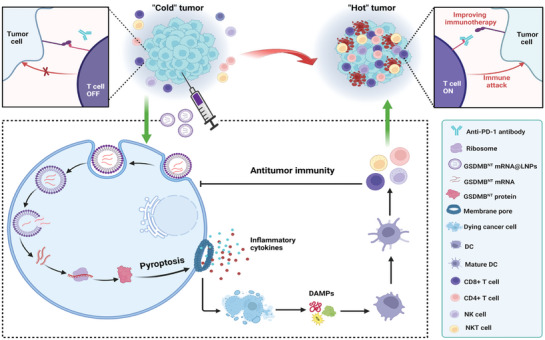
Schematic of antitumor immunity via GSDMB^NT^ pyroptosis and characterization of GSDMB^NT^ mRNA@LNP‐mediated mRNA@LNPs. Intratumoral injection of mRNA lipid nanoparticles encoding the N‐terminal domain of GSDMB can trigger pyroptosis, induce antitumor immunity, and promote αPD‐1‐mediated immunotherapy. Reproduced with permission.^[^ ^95^ ^]^ Copyright 2023, Springer Nature.

At present, few studies have investigated GSDMB protein‐mediated pyroptosis. Several studies have indicated that GZMA can activate GSDMB protein‐mediated pyroptosis. However, the function of the GSDMB protein in tumor progression remains undetermined. Some researchers argue that the GSDMB protein may promote tumor cell proliferation because of its high expression in certain tumors. On the other hand, researchers believe that when activated in tumor cells with high GSDMB expression, pyroptosis can stimulate antitumor immunity and eradicate solid tumors. Xu et al.^[^ ^95^ ^]^ introduced a strategy for single‐molecule messenger RNA (mRNA) nanomedicine that utilizes N‐terminal mRNA lipid nanoparticles (LNPs) specifically designed to encode gasdermin‐B, aiming to induce pyroptosis and activate a strong antitumor immune response. Experiments with several female mouse models revealed that mRNA/LNP‐induced pyroptosis converts cold tumors into hot tumors, thereby establishing a beneficial feedback mechanism that enhances antitumor immunity. Moreover, pyroptosis triggered by mRNAs/LNPs enhanced the sensitivity of tumors to αPD‐1 immunotherapy, leading to inhibited tumor growth and prevention of metastasis. The single‐agent mRNA nanomedicine introduced in this study has the dual function of directly targeting and killing tumor cells, and of inducing robust and safe antitumor immunity through the use of single‐drug mRNA. Moreover, by directly expressing the N‐terminus of the GSDM protein, this approach obviates the necessity for protease cleavage. The rapid and effective induction of pyroptosis can be combined with immune checkpoint inhibitors, thereby enhancing the overall efficacy of immunotherapy. This single‐agent mRNA nanomedicine therapy is both straightforward and efficient, demonstrating significant potential for clinical translation (**Figure** **12**). Teng et al.^[^ ^96^ ^]^ developed a bispecific antibody (IBI315) that was bound to a recombinant form of human recombinant lmmunoglobulin G1 (IgG1), facilitating the targeting of both PD‐L1 and human epidermal growth factor receptor 2 (Her2) and studied its effectiveness against tumors and the related mechanisms. This innovative antibody facilitated the binding of Her2‐positive cancer cells to PD‐1‐positive T cells, which resulted in notably enhanced antitumor responses in tumor‐bearing mice, patient‐derived xenograft model mice, and organoids with human immune components, both in vitro and in vivo, in comparison with single maternal antibodies or their combinations. Additionally, treatment with IBI315 promoted the recruitment and activation of immune cells within tumors. Mechanistically, IBI315 induced pyroptosis mediated by GSDMB, leading to the recruitment and activation of T cells. These activated T cells produced Interferon‐gamma (IFNγ), increased GSDMB expression, and created a positive feedback mechanism linking T‐cell activation to tumor cell destruction. Importantly, elevated levels of GSDMB were observed in Her2‐positive gastric cancer cells, supporting the theoretical rationale for the efficacy of IBI315. Preclinical investigations indicate that IBI315 holds promise as an immunotherapy for patients with Her2‐positive gastric cancer through the application of bispecific antibodies, thereby broadening treatment opportunities for this group.

The current findings concerning pyroptosis mediated by the GSDMB protein and that mediated by the GSDMA protein are analogous, as both emphasize the upregulation of target proteins and precise targeted activation.

### 3.5 GSDMC‐Targeting Biomaterials for Pyroptosis and Antitumor Immunotherapy

**Figure 13:**
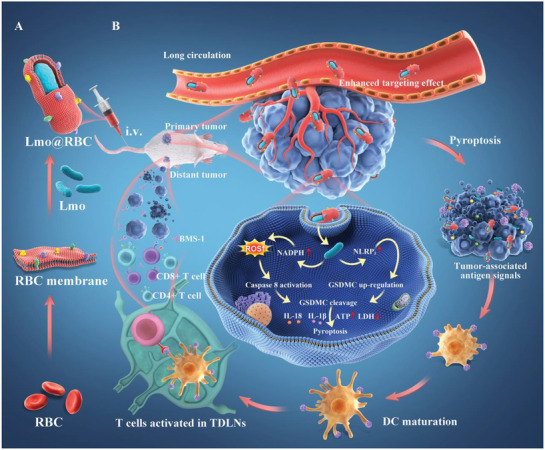
Schematic diagram of Lmo@RBC antitumor immunotherapy inducing pyroptosis. A) Schematic illustration of the extraction of RBC membranes and the preparation of Lmo@RBC. B) Lmo @ RBC can effectively target tumors after intravenous administration, trigger pyroptosis of cancer cells and release pro‐inflammatory substances, and induce DCs maturation and T cell activation in tumor‐draining lymph nodes (TDLNs), thereby effectively inhibiting primary tumors and distal tumors. Reproduced with permission.^[^ ^97^ ^]^ Copyright 2022, American Chemical Society.

The GSDMC, like GSDMB, is a member of the gasdermin family that has received comparatively less attention in recent research. Its role involves inducing pyroptosis through caspase‐8 activation. Consequently, the potential of GSDMC as a target for tumor treatment remains relatively understudied. This is mainly due to the requirement for strong caspase‐8‐activating conditions, which necessitate a certain concentration of TNFα to stimulate cancer cells. Additionally, there is controversy surrounding the use of GSDMC as an antitumor target, with some researchers suggesting that it may actually promote tumor proliferation. To address these challenges, Shen et al.^[^ ^97^ ^]^ designed a novel immunotherapy approach that involved encapsulating *Listeria monocytogenes* (Lmo) in natural red blood cell (RBC) membranes while effectively removing specific virulence factors (referred to as Lmo@RBC). This biomimetic construct exhibited prolonged circulation in the bloodstream and responded to hypoxic conditions within tumors, thereby supporting anaerobic colonization by Lmo. Consequently, Lmo@RBC not only triggered a systemic inflammatory response but also promoted greater accumulation of Lmo within tumor tissues. A genome screening analysis of tumors subjected to intravenous administration of phosphate‐buffered saline (PBS), Lmo, or Lmo@RBC revealed for the first time that treatment with Lmo@RBC prompted extensive pyroptosis in a manner dependent on the pore‐forming protein GSDMC, leading to the alleviation of the immunosuppressive tumor microenvironment. As a result, a robust, systemic, and lasting antitumor immune response was stimulated, yielding significant therapeutic benefits against solid tumors and their metastasis (**Figure** **13**).

The activation strategy for GSDMC protein‐mediated pyroptosis remains relatively straightforward. Current research indicates that TNFα is the sole identified activator of the GSDMC protein. Therefore, it is essential to investigate additional potential activation mechanisms for GSDMC. Moreover, a critical focus of research at present is developing methods to facilitate the direct transport of TNFα, which is prone to rapid decomposition in the body, to target tumor cells.

## 4 Activation of Pyroptosis by Upregulating GSDM Protein Expression

**Figure 14:**
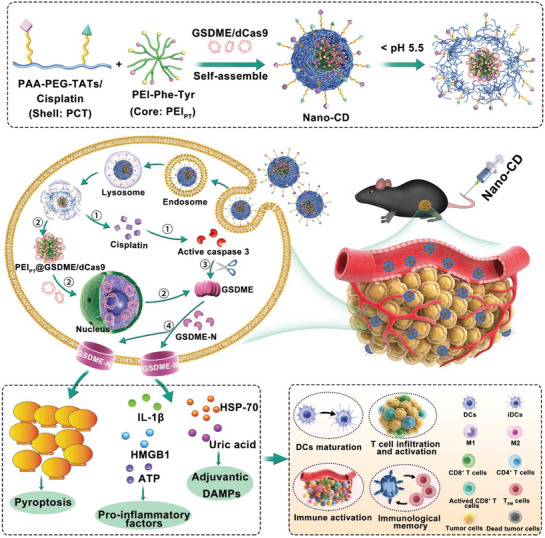
Nano‐CD scaffold preparation process and induction of pyroptosis trigger antitumor immune schematic diagram. Under acidic conditions in cells, Nano‐CD scaffold releases cisplatin and CRISPR/dCas9 plasmid. Under the action of CRISPR/dCas9, the endogenous GSDME protein supplied by tumor cells is cleaved by cisplatin‐induced activated caspase‐3, inducing strong pyroptosis, thereby promoting antitumor immunity. Reproduced with permission.^[^ ^103^ ^]^ Copyright 2023, Springer Nature.

**Figure 15:**
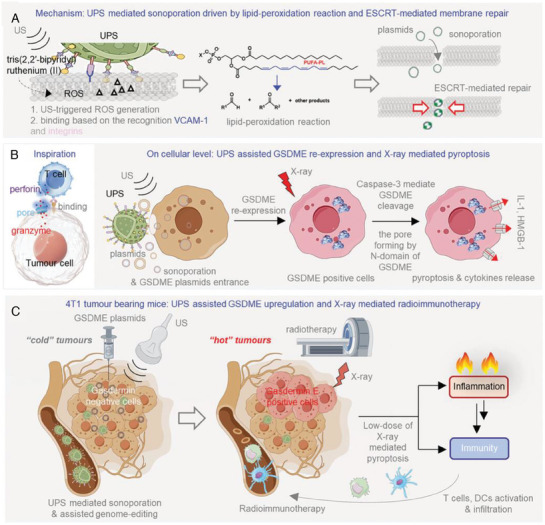
Schematic representation of antitumor immunity triggered by UPS‐assisted GSDME re‐expression combining with low‐dose X‐ray‐mediated pyroptosis. A), Mechanism of UPS‐mediated sonoporation mediating transient pores in cell membrane. B), Schematic diagram of UPS assisting gene editing plasmid to enter cells under ultrasound to up‐regulate GSDME protein and induce pyroptosis under radiotherapy. C), In vivo reprogramming of gsdm‐negative 4T1 tumor‐bearing mice with ups‐assisted GSDME reexpression, and activation of antitumor immunotherapy using X‐ray‐induced pyroptosis. Reproduced with permission.^[^ ^120^ ^]^ Copyright 2024, Wiley‐VCH.

Pyroptosis was initially observed in macrophages following bacterial infection or exposure to bacterial toxins, and it was initially mistaken as a type of caspase‐1‐dependent macrophage‐specific cell death that cleaves the proinflammatory cytokine IL‐1β. However, in 2015, two independent studies identified GSDMD as the key player in pyroptosis. Dixit et al.^[^ ^17a^ ^]^ conducted a chemical mutagenesis screen of mouse mutants and reported that GSDMD is involved in the activation of inflammasomes via the noncanonical pathway induced by LPS. Shao et al.^[^ ^17d^ ^]^ employed CRISPR‐Cas9 technology to conduct a comprehensive genome‐wide screening, leading to the important discovery that GSDMD functions as the substrate for all inflammatory caspases and serves as the definitive executor of pyroptosis. These findings illuminate the underlying molecular mechanisms associated with pyroptosis, effectively establishing gasdermin proteins as pivotal mediators of programmed cell death. While the GSDM protein ultimately orchestrates pyroptosis, numerous cancer cells either do not express the GSDM protein or express it at notably low levels due to their adaptive immune evasion strategies. To broaden therapeutic strategies aimed at inducing pyroptosis in tumors, researchers are increasingly directing their efforts toward methods that increase GSDM protein expression within cancer cells. Additionally, studies have focused on the delivery of the GSDM protein or its activated N‐terminus as a means to trigger GSDM‐mediated pyroptosis. This approach not only aims to circumvent the challenges posed by low GSDM protein levels in cancer cells but also aims to exploit the pyroptotic pathway as a novel avenue for effective cancer treatment. Sun et al.^[^ ^98^ ^]^ developed a novel prodrug nanomicelle known as AOZN, which is designed to exploit the unique characteristics of the tumor microenvironment. This innovative formulation incorporates γ‐oryzanol (Orz) as an epigenetic modulator, α,β‐methylene adenosine 5′ diphosphate (AMPCP) as an adenosine inhibitor, and a GSH‐activated cross‐linking agent. In the TME, which is characterized by elevated levels of glutathione, AOZN effectively triggers the release of both Orz and AMPCP. The introduction of Orz is associated with the upregulation of GSDMD expression. Concurrently, AMPCP increases the levels of ATP, which subsequently activates caspase‐1. The activation of caspase‐1 leads to the cleavage of GSDMD, facilitating pyroptosis in tumor cells. This mechanism not only contributes to tumor cell death but also demonstrates the strong synergistic effect between Orz and AMPCP in the context of alleviating the immunosuppressive TME. Moreover, Orz plays a critical role in increasing the expression of PD‐L1, which is important in the context of immune evasion by tumor cells. By increasing PD‐L1 expression, Orz increases the sensitivity of tumors to treatments targeting PD‐L1. Consequently, AOZN represents a promising strategy to inhibit tumor growth, increase therapeutic response rates to PD‐L1 treatments, and ultimately prolong the survival of melanoma‐bearing mice. Zhang et al.^[^ ^100^ ^]^ reported that the low‐dose epigenetic agent decitabine can significantly increase the expression level of GSDME in RM‐1 cells, a prostate cancer cell line. This increase is crucial, as it promotes a shift from traditional apoptosis—a type of programmed cell death—towards pyroptosis, a form of inflammatory cell death, particularly following treatment with PDT. This observation highlights the potential of decitabine as a therapeutic enhancer in the context of PDT. In addition to these findings, researchers have innovated a dual‐responsive nanomedicine, referred to as TSD@LSN‐D, which is engineered to deliver αPD‐L1. This breakthrough demonstrates promising synergistic effects that are key for advancing cancer immunotherapy strategies. Notably, in the RM‐1 prostate cancer model, which is characterized by low immunogenicity, TSD@LSN‐D was shown to elicit a robust antitumor immune response. This immune activation not only effectively suppresses the growth of primary tumors but also contributes to the establishment of long‐lasting immune memory, which is crucial for preventing the recurrence and metastasis of prostate cancer. Dai et al.^[^ ^105^ ^]^ proposed an innovative epigenetic approach to initiate cancer pyroptosis in conjunction with radiotherapy. They created a nanoligand (PWE) by utilizing metal‒phenol coordination among a polyphenol DNA methyltransferase inhibitor (epigallocatechin‐3‐gallate, EGCG), a high‐Z radiosensitizer (W^6+^), and block copolymers modified with polyphenols. EGCG increased the expression of GSDME, whereas PWE facilitated the cleavage of GSDME into a fragmented N‐terminus via calpain I induced by radiotherapy. PWE strengthened the immune response associated with conventional radiotherapy, decreased the number of regulatory T cells, and provided new perspectives on tumor radiotherapy. Gong et al.^[^ ^103^ ^]^ described a synergistic nano‐CRISPR scaffold known as Nano‐CD, which promoted the expression of endogenous GDSME through the use of specific small guide RNA (sgRNAs) identified via functional screening while also releasing cisplatin to trigger immune cell apoptosis. The amplified antitumor immune response resulted from the lytic characteristics of the intracellular material and the increased expression of GDSME, subsequently causing pyroptosis and the liberation of tumor‐associated antigens. Individually, these elements are insufficient for effective tumor control; however, the concurrent action of cisplatin and the autogenous supply of GSDME leads to a significant reduction in tumor growth in both primary and recurrent melanoma models. When used in conjunction with immune checkpoint inhibitors, Nano‐CD promoted durable immune memory, fostered a robust systemic antitumor immune response, curtailed disease recurrence, mitigated lung metastasis, and increased survival rates in mice with melanoma (**Figure** **14**). Wang et al.^[^ ^120^ ^]^ developed a user‐friendly ultrasound (US)‐controlled perforation system (UPS) with an extended shelf life to improve the delivery of free genome manipulators. This UPS can precisely infiltrate the membranes of tumor cells through controlled lipid peroxidation without compromising cell viability. In vitro experiments demonstrated that plasmids with transmembrane properties can penetrate cells and facilitate genome editing through the aid of the UPS, achieving an efficiency of up to 90%. In vivo studies revealed that this UPS possessed biodegradability, nonimmunogenicity, and tumor‐targeting capabilities, enabling it to kill tumor cells when subjected to US. Using UPS‐assisted genome editing, the expression of gasdermin E was effectively restored in 4T1 tumor‐bearing mice, achieving pyroptosis‐mediated antitumor immunotherapy through low‐dose X‐ray irradiation. The methodology proposed in this study for enhancing plasmid entry into cells for gene editing through ultrasound‐mediated controllable lipid peroxidation offers novel insights for the design of safe and efficient genome editing auxiliary tools. Furthermore, it offers valuable insight into the activation of pyroptosis‐induced anti‐tumor immunotherapy in tumor cells with low expression of the GSDM protein (**Figure** **15**).

Currently, upregulating the expression of proteins related to the GSDM family is an effective strategy for inducing pyroptosis. Various methods, including drugs and gene editing, can effectively increase the expression of these proteins; however, strategies that efficiently and specifically upregulate them require further optimization.

## 5 Conclusion and Prospects

Pyroptosis is a well‐studied form of ICD, and increasing attention has been given to GSDM family proteins and their activation mechanisms since their identification as the final execution proteins of pyroptosis by Shao et al. in 2015.^[^ ^7^ ^]^ As the understanding of pyroptosis has increased, the relationship between pyroptosis and the immune response has also been explored. Notably, even a small amount of cancer pyroptosis can potentiate strong antitumor immune response for solid tumors.^[^ ^8a^ ^]^ The use of biomaterials represents an effective solution to the safety issues that arise from excessive and off‐target pyroptosis, due to their high degree of specificity and precise targeting capabilities. A variety of biomaterials, including liposomes, metal nanomaterials, polymer materials, and cell membrane materials, utilize their particle size and surface‐specific targeting antibodies or molecules to effectively target tumor sites, thereby reducing damage to normal tissues in comparison to traditional chemotherapy. Moreover, biomaterials designed with endogenous responses, such as pH response, glutathione response, and hydrogen peroxide response, based on the characteristics of the tumor microenvironment, enhance the specificity of pyroptosis activation. Furthermore, exogenously activated biomaterials, including photothermal materials, photodynamic materials, and ultrasound‐responsive materials, can be employed in conjunction with relevant diagnostic techniques to specifically induce pyroptosis at the tumor site, thereby markedly reducing the safety risks associated with excessive and off‐target effects. Furthermore, biomaterials provide an effective solution to the challenge posed by immune‐escaping tumor cells, which exhibit low expression of GSDM family proteins, thereby making it difficult to induce pyroptosis. Biomaterials can effectively enhance the expression of GSDM family proteins in tumor cells by facilitating the targeted delivery of drugs (such as DAC), GSDM family proteins, and mRNA to these cells, thereby promoting the subsequent induction of pyroptosis. Here, we focus on the GSDM protein‐targeting pyroptosis‐inducing biomaterials and review various initiating‐strategies, such as PDT, PTT, bacterial and oncolytic virus therapy, and genetic engineering therapy, that can induce pyroptosis‐mediated antitumor immunity. However, there are still many issues that need to be resolved before strategies inducing pyroptosis can be applied to the clinical treatment of cancer.

### 5.1 Challenges in Activating Pyroptosis

One drawback of pyroptosis in antitumor immunotherapy is the low or absent expression of the GSDM protein, which is a mechanism of immune escape employed by tumor cells. Although researchers have attempted to increase GSDM protein expression in tumor cells via the small‐molecule drug decitabine or gene editing methods, the effectiveness and safety of these approaches still require improvement.^[^ ^21a^ ^]^ Decitabine, when used to upregulate GSDM protein expression in tumor cells, has low upregulation efficacy and poor targeting ability, which can trigger systemic inflammatory responses. Thus, it is imperative to discover more effective and targeted drugs for GSDM protein regulation in tumor cells. Furthermore, upregulating GSDM protein expression in tumor cells through gene editing poses challenges.^[^ ^95^, ^99^ ^]^ The components necessary for gene editing, such as the Cas9 protein coding sequence and corresponding guide RNA (gRNA), may exceed the loading capacity of commonly used vectors (e.g., adeno‐associated virus (AAV)).^[^ ^121^ ^]^ This limitation hampers the effective delivery of large vectors. Moreover, the transduction efficiency of different types of vectors (e.g., viral or nonviral) varies across cell types and tissues, which may result in imprecise targeting of desired cells, leading to nonspecific editing.^[^ ^122^ ^]^ Additionally, gene editing technology has the risk of causing unintentional mutations at nontarget sites. Notably, the use of viral vectors may elicit an immune response from the host, resulting in rejection of the vector or edited cells, particularly with repeated administration.^[^ ^123^ ^]^ Hence, developing a more effective and safer vector for gene editing and GSDM protein regulation in tumor cells is crucial.

### 5.2 Challenges for Pyroptosis‐Inducing Biomaterials

While the strong immunogenicity of pyroptosis offers significant advantages in immunotherapy, the issue of uncontrolled or excessive pyroptosis leading to an inflammatory storm remains.^[^ ^6^ ^]^ Effectively controlling the extent of pyroptosis has therefore emerged as a major problem, posing a challenge to the research and development of related biomaterials. To address this, it is crucial to design biomaterials with improved targeting capabilities to minimize accumulation in nontumor cells, given that the expression of the GSDM protein is typically greater in normal cells than in tumor cells. Poor targeting can easily trigger an inflammatory cytokine storm. Additionally, optimizing the response mechanisms of these materials is essential. Compared with endogenous response methods (e.g., pH, GSH, and H_2_O_2_), exogenous response methods (e.g., light, ultrasound, and radiation) demonstrate superior specificity. However, because most tumors are located deep within tissues, greater consideration should be given to penetration depth and the design of integrated diagnostic and treatment materials for locating and treating the tumor using exogenous response methods.

### 5.3 Challenges in the Clinical Translation of Strategies that Induce Pyroptosis

The primary challenge hindering the clinical translation of strategies that induce pyroptosis is the safety of biomaterials that promote this form of cell death. Currently, biomaterials known to induce pyroptosis include liposomes, polymers, inorganic nanomaterials, metal‒organic frameworks, and engineered bacteria and viruses, among others. The biological safety of these materials requires further validation. Additionally, pyroptosis is characterized as a highly immunogenic form of cell death, and the excessive nontargeted induction of pyroptosis can provoke a substantial inflammatory response within the body. Therefore, it is crucial to consider the method of induction when designing clinical treatments. For the successful clinical translation of pyroptosis, several key steps are needed: first, it is essential to explore the mechanisms underlying pyroptosis and identify new activation targets, with the aim of discovering more specific activation methods; second, the integration of gene editing technology with pyroptosis should be advanced, as this combination could enable personalized treatment with strategies that induce pyroptosis; finally, the development of targeted drugs that can accurately induce pyroptosis by specifically targeting tumor cells is necessary, as this will have a great impact on the clinical application of treatments that induce pyroptosis.

## Conflict of Interest

The authors declare no conflict of interest.

## Author Contributions

H.Y., T.C., X.H., and W.Z. contributed equally to this work. Q.W., H.Z., and L.L. are co‐corresponding authors with the review. Q.W., H.Z., and L.L. conceived the review. Q.W. wrote the manuscript with inputs from all authors. All authors discussed the manuscript.
